# Are low-value care measures up to the task? A systematic review of the literature

**DOI:** 10.1186/s12913-016-1656-3

**Published:** 2016-08-18

**Authors:** Eline F. de Vries, Jeroen N. Struijs, Richard Heijink, Roy J. P. Hendrikx, Caroline A. Baan

**Affiliations:** 1 Department Tranzo (Scientific Center for Care and Welfare), Tilburg University, Tilburg School of Social and Behavioral Sciences, P.O. Box 90153, 5000 LE Tilburg, The Netherlands; 2 Department of Quality of Care and Health Economics, National Institute of Public Health and the Environment (RIVM), Center for Nutrition, Prevention and Health Services, P.O. Box 1, 3720 BA Bilthoven, The Netherlands

**Keywords:** Low-value care, Measures, Quality improvement, Performance measures

## Abstract

**Background:**

Reducing low-value care is a core component of healthcare reforms in many Western countries. A comprehensive and sound set of low-value care measures is needed in order to monitor low-value care use in general and in provider-payer contracts. Our objective was to review the scientific literature on low-value care measurement, aiming to assess the scope and quality of current measures.

**Methods:**

A systematic review was performed for the period 2010–2015. We assessed the scope of low-value care recommendations and measures by categorizing them according to the Classification of Health Care Functions. Additionally, we assessed the quality of the measures by 1) analysing their development process and the level of evidence underlying the measures, and 2) analysing the evidence regarding the validity of a selected subset of the measures.

**Results:**

Our search yielded 292 potentially relevant articles. After screening, we selected 23 articles eligible for review. We obtained 115 low-value care measures, of which 87 were concentrated in the cure sector, 25 in prevention and 3 in long-term care. No measures were found in rehabilitative care and health promotion. We found 62 measures from articles that translated low-value care recommendations into measures, while 53 measures were previously developed by institutions as the National Quality Forum. Three measures were assigned the highest level of evidence, as they were underpinned by both guidelines and literature evidence. Our search yielded no information on coding/criterion validity and construct validity for the included measures. Despite this, most measures were already used in practice.

**Conclusion:**

This systematic review provides insight into the current state of low-value care measures. It shows that more attention is needed for the evidential underpinning and quality of these measures. Clear information about the level of evidence and validity helps to identify measures that truly represent low-value care and are sufficiently qualified to fulfil their aims through quality monitoring and in innovative payer-provider contracts. This will contribute to creating and maintaining the support of providers, payers, policy makers and citizens, who are all aiming to improve value in health care.

**Electronic supplementary material:**

The online version of this article (doi:10.1186/s12913-016-1656-3) contains supplementary material, which is available to authorized users.

## Background

The concept of low-value care, defined as services that provide no benefit to patients or can even cause harm [1], has received much attention in recent years in Western countries. Reducing the use of low-value care is expected to contribute to cost containment and more efficiency in health care [2–4]. It leads to a reduction in medical spending without harming health outcomes and it may stimulate a reallocation of resources to high-value services [3]. In this way, measuring low-value care for which the non-effectiveness is proven provides information on a specific type of inefficiency, i.e. spending with no benefit, which can be used besides other, more indirect, types of efficiency analysis such as traditional cost-effectiveness studies or analyses of practice variation.

Internationally, several initiatives have been launched to reduce low-value service utilization, among which the Choosing Wisely (CW) campaign in the US. Similar initiatives have originated in 12 other countries including the United Kingdom, Canada, Australia and the Netherlands [3, 5]. In the CW campaign, participating specialty societies produce lists of recommendations that are to be discussed in the doctor’s office, as for example, ‘don't order diagnostic tests at regular intervals (such as every day), but rather in response to specific clinical questions’ [6]. Ideally, these lists of recommendations would meet the CW criteria: 1) each of the services is within the specialty’s purview, 2) each of the services is frequently used or costly, 3) each recommendation is based on sufficient evidence, and 4) the process for developing the recommendation list is documented and is made available to the public if requested [7]. In general, the recommendations aim to increase awareness among both doctors and patients [4] and subsequently influence the decision whether or not to use a specific service.

Besides these rather generic *recommendations*, studies have tried to assess the prevalence and geographic or practice variation in low-value care utilization (e.g. [8–11]) using direct *measures* of low-value care. The aim of the direct measures differs from the aim of recommendations. Where recommendations aim to create awareness among physicians and patients, low-value care measures may be widely used, for example in payer-provider contracts [12, 13] and for monitoring low-value care initiatives [3, 14].

To meet these aims, low-value care measures need to be methodologically sound [15, 16]. Otherwise, using these measures might create misinterpretation, underuse of indicated services, patient selection or damage the patient-physician relationship [17]. To date, only one study [18] reviewed the state of low-value care measurement by performing a scan of the published and grey literature. They found 37 specified measures and 123 services that may be developed into measures, covering mainly diagnostic or therapeutic areas. Furthermore, another study [19] identified a set of low-value services and demonstrated significant variance in its utilization between hospital referral regions in the US.

Still, major knowledge gaps exist in the literature on measuring low-value care. First, there is lack of knowledge regarding the validity of current low-value care measures [15, 16]. As Baker et al. [14] pointed out earlier, low-value care measures must at least be rigorously evidence-based. In addition, they must be able to detect variation between providers, regions or countries, reflect actual cases of the concept of interest, be supported by correlations to other measures indicating the same concept, and not be subject to substantive systemic bias (i.e. importance, coding or criterion validity, construct validity and risk adjustment) [20]. Therefore, specific standards for how to develop and assess low-value care measures should be developed [14, 17]. Second, it is unclear whether current low-value care measures cover the whole continuum of care. This is important, because it was argued that low-value care use is present in all sectors along the care continuum [14, 21]. However, the low-value service recommendations from the CW initiative cover mainly specialist care in the cure sector [7].

In this study, we aimed to start filling these gaps by performing a systematic review of the recent scientific literature on low value care measurement. Our objective was twofold. Firstly, to assess the scope of low-value care *recommendations* and *measures* in the literature by categorizing them according to health care function (such as curative care, long-term care and rehabilitation). Secondly, to assess the quality of the *measures* by 1) analysing their development process and the evidence that underlies the measures and 2) analysing the evidence regarding the validity of a selection of the included measures.

## Methods

### Study design and search strategy

A systematic review of the literature was performed, focusing on English-language articles published between January 2010 and January 2015. As recommended by Cochrane [22], we performed our search in multiple databases including EMBASE, Medline, SciSearch, BIOSIS Previews and GLOBAL Health. We developed a search strategy to identify articles matching a variation of the following search terms: 1) initiatives, design, measuring, indicators, instrument, identifying, index; 2) waste, overuse, overutilization, misuse, low-value; and 3) health care, cure, care, prevention. Additional file 1 gives a detailed description of the search strategy.

### Article selection

Two researchers (EFdV & RJPH) independently reviewed the relevance of the articles by screening titles and abstracts. As recommended by Cochrane [22], we included articles from peer-reviewed journals only. The full-text was retrieved when both researchers considered the paper relevant. Articles were eligible for review when they met the following predefined criteria: 1) the low-value service recommendation or measure in the paper matched the definition ‘services that provide no benefit to patients or may even cause harm [1]’; 2) the low-value service recommendation or measure was described using clinical details such as diagnosis, patient population and treatment. We removed duplicate articles and replies or commentaries and theoretical or discussion articles that did not present any low-value service recommendations or measures. Any disagreement between the reviewers was resolved by discussion and consensus.

### Data extraction

We extracted general characteristics of the articles (i.e. name of first author, year of publication, country, aim of the paper, methods) and the measures (i.e. the name of the measure, the numerator, the denominator, exclusion criteria and direction). In addition, we retrieved the original source or reference of the measure.

### Recommendations versus measures

The literature search yielded both recommendations and measures for low-value care. We considered a description of low-value care as ‘measure’ when at least a numerator and denominator were specified as such. We identified the scope of both recommendations and measures, while the quality assessment was performed for the measures only.

### Categorizing low-value care recommendations and measures by function in health care

All recommendations and measures were categorized using the Classification of Health Care Functions (ICHA-HC) as defined by the Organization for Economic Co-operation and Development (OECD), the World Health Organization (WHO) and Eurostat [23]. The ICHA-HC provides a framework to classify services according to their purpose or function and is commonly used to compare medical services internationally. It covers the entire continuum of the health system, i.e. curative care, rehabilitative care, long-term care and preventive care. We subcategorized curative care into general (i.e. primary) care and specialized care. General care involves basic care such as routine examinations, basic maternity care, routine diagnosis and follow-up, prescriptions and vaccinations (unless they are covered under a preventive program) [23]. Specialized care involves more complex technology and is often a breakdown from the basic fields (e.g. neurosurgery or allergology) [23]. In addition, the measures were categorized according to the non-functional categories ancillary services (i.e. laboratory, imaging, transport), and medical goods (i.e. pharmaceutical and therapeutic appliances).

### Assessing the quality of low-value care measures

We assessed the quality of the measures by 1) analysing their development process and the level of evidence underlying the measures, and 2) analyse the validity of a selection of the measures.

#### Development process and level of evidence

We distinguished two groups: A) articles that translated low-value service recommendations into low-value care measures, and B) articles that used measures previously developed by institutions. For both groups we reviewed how the measures were developed.

For group A, we searched for evidence underlying the recommendations. We categorized each measure based on the evidence, distinguishing three levels of evidence: 1) a combination of evidence from the literature (trial or review), guidelines and from CW, United States Preventive Services Task Force (USPSTF) or National Institute of Clinical Excellence (NICE) recommendations, 2) evidence from the literature (trial or review) or guidelines, and 3) evidence not found. As criteria for developing CW recommendations do not prescribe the level of evidence required [7] we labelled measures with CW, USPSTF or NICE evidence only, as ‘unknown’. We valued the first level highest, and the third level lowest.

For group B, we distinguished the same levels of evidence. However, here we specifically searched for elements of a quality label indicating the soundness of the measure. A National Quality Forum (NQF) endorsement corresponds with the qualification of ‘minor or no evidence gaps’ [20]. Measures with such qualification have the strongest evidence base regarding importance, face validity, criterion validity, construct validity and risk adjustment [20]. Therefore, NQF endorsed measures were valued highest. The Agency of Healthcare Research and Quality (AHRQ) and the Centers of Medicare and Medicaid Services’ (CMS) Quality provide information on the level of evidence by specifying the literature underpinning the measure. Therefore, measures from these sources were valued second best.

For both groups, our assessment was limited to the evidence provided in the reviewed article and the first document retrieved by reference tracking.

#### Validity

We selected a subset of five unique measures in order to gain insight in the quality of the low value care measures. Ideally, we would extensively assess each measure regarding their validity. However, for 115 measures this was beyond the scope of this review. Therefore, we chose five unique measures that appeared most frequently in the reviewed articles, assuming more information on validity to be available for these measures. For these five measures, we searched for evidence regarding the measures’ validity by reviewing the original source and reference tracking. In addition, we performed a PubMed search using key words from the name of the measures (i.e. diagnosis and procedure) and “low-value” or “overuse”, augmented with “validity”. Specifically, we searched for studies that aimed to assess the validity of the selected low-value care measures. Hereby, we distinguished between the most commonly used types of validity (as seen in e.g. [20, 24, 25]): face validity, coding/criterion validity (i.e. reflect actual cases low-value care) and construct validity (i.e. supported by correlations to other measures indicating low-value care) [20]. Face validity refers to the empirical or clinical rationale of the measure, and therefore we used the information from Table 2 for this criterion.

## Results

### Article retrieval

**Fig. 1:**
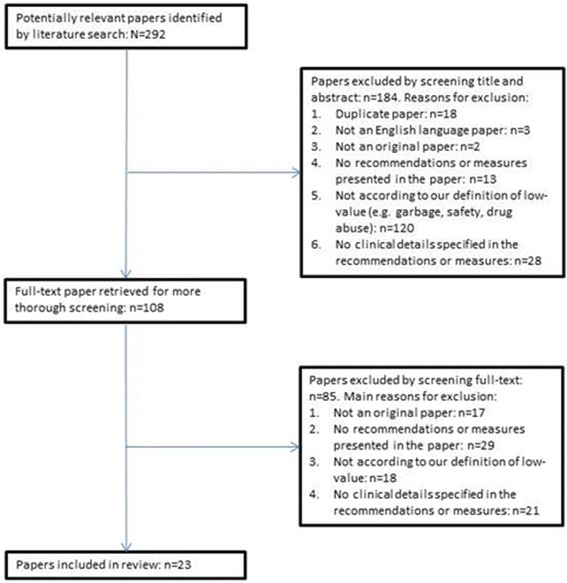
Flow chart summarizing article selection

Our literature search yielded 292 potentially relevant articles (Fig. 1). Based on titles and abstracts, 108 articles were selected for full-text retrieval and thorough screening. This screening process generated 23 articles that were eligible for review. Main reasons for exclusion were using a different definition of low-value care (*n =* 138), for example articles on garbage, patient safety or drug abuse, or not providing clinical details (*n =* 49). Figure 1 shows all reasons for exclusion.

### Article characteristics

All articles were published after 2011 and the vast majority of the 23 included articles originated from the United States (*n =* 22) (Table 1). Seven articles explicitly focused on low-value care measures. One of these reviewed the literature on low-value care measurement [18], and six were empirical studies measuring low-value care utilization [2, 8, 10, 11, 19, 26]. Low-value care recommendations were presented in 17 articles of which most were related to the CW campaign (*n =* 12).

**Table 1:** General characteristics of the included articles (*n =* 23)

First author	Year of publication	Country	Aim	Method	Number retrieved		Recommendation initiative
					Measures^a^	Recommendations	
AGS Choosing Wisely AGSCW Workgroup [7]	2013	US	To identify five services that physicians and patients should question.	Review + Delphi/consensus	0	5	CW
AGS Choosing Wisely Workgroup [34]	2014	US	To identify another five services that physicians and patients should question.	Delphi/consensus	0	5	CW
Amos [35]	2015	US	To determine the prevalence of PIMs for older adults in Elimia-Romagna, Italy, using updated Maio criteria.	Empirical analysis	0	16	Other
Bulger [36]	2013	US	To identify five services that physicians and patients should question.	Review + Delphi/consensus	0	5	CW
Chan [18]	2013	US	To describe and critique the current state of overuse measurement.	Review	37	122	Other
Colla [8]	2015	US	To develop claims-based algorithms to estimate the prevalence of Choosing Wisely services and to examine the demographic, health and health care system correlates of low-value care at a regional level.	Empirical analysis	11	0	N.A.
Elshaug [37]	2012	AUS	To develop and apply a novel method for scanning a range of sources to identify existing health care services (excluding pharmaceuticals) that have questionable benefit, and produce a list that warrant further investigation.	Review	0	174	Other
Halpern [38]	2014	US	To present the Critical Care Societies Collaborative top 5 list in Critical Care Medicine and describe its development.	Review + Delphi/consensus	0	5	CW
Hicks [39]	2013	US	To identify five services that physicians and patients should question.	Review + Delphi/consensus	0	5	CW
Kale [26]	2013	US	The objective of this study was to determine whether the overuse and misuse of health care services in the ambulatory setting has decreased in the past decade.	Empirical analysis	13	0	N.A.
Keyhani [40]	2013	US	To compare rates of overuse in different health care systems and examine whether certain systems of care or insurers have lower rates of overuse of health care services.	Systematic review	0	7	Other
Korenstein [41]	2012	US	To perform an extensive search for studies of overuse of therapeutic procedures, diagnostic tests, and medications in the United States and describe the state of the literature.	Extensive search	0	33	Other
Mathias [10]	2012	US	To characterize performance on imaging-use measures, determine whether performance was consistent across measures, and identify hospital characteristics associated with highest-decile imaging use.	Empirical analysis	4	0	N.A.
Morden [11]	2014	US	To measure the prevalence and describe the geographic variation of short-interval (repeated in under 2 years) DXAs among Medicare beneficiaries and estimated the cost of this testing and its responsiveness to payment change.	Empirical analysis	4	0	N.A.
Onuoha [42]	2014	US	To develop a top 5 list of unnecessary medical services in anesthesiology.	Review + Delphi/consensus	0	5	CW
Quinonez [43]	2013	US	To produce top 5 lists.	Review + Delphi/consensus	0	5	CW
Rouster-Stevens [44]	2014	US	To create a pediatric rheumatology Top 5 list as part of the American Board of Internal Medicine Foundation’s Choosing Wisely campaign.	Review + Delphi/consensus	0	5	CW
Schuur [45]	2014	US	To create a top-five list of tests, treatments, and disposition decisions that are of little value, are amenable to standardization, and are actionable by emergency medicine clinicians.	Delphi/consensus	0	5	CW
Schwartz [2]	2014	US	To develop claims-based measures of low-value services, examine service use (and associated spending) detected by these measures in Medicare, and determine whether patterns of use are related across different types of low-value services.	Empirical analysis	26	0	N.A.
Segal [19]	2014	US	To identify a set of possible indicators of overuse that can be operationalized with claims data and to describe variation in these indicators across the hospital referral regions (HRRs).	Empirical analysis	20	0	N.A.
Wiener [46]	2014	US	To create a top 5 list.	Review + Delphi/consensus	0	5	CW
Williams [47]	2012	US	To present the final five Choosing Wisely Don’t do recommendations, the rationale for these specific recommendations, and two other recommendations.	Delphi/consensus	0	5	CW
Wood [48]	2013	US	To report on the CW top 5 list.	Review + Delphi/consensus	0	5	CW

*AGS* American Geriatrics Society, *AUS* Australia, *CW* Choosing Wisely, *N.A.* Not Applicable, *PIM* Potentially Inappropriate Medications, *US* United States

^a^at least a numerator and denominator was specified

Our search yielded 115 low-value care measures and 412 low-value care recommendations. Additional file 2 shows the characteristics of the 115 low-value care measures (i.e. containing a numerator and denominator). Out of these 115 measures, 42 contained exclusion criteria. For one of these measures (measure no. 72, Additional file 2), the direction of the measure was specified. Additional file 3 lists all recommendations (i.e. not containing numerator and/or denominator).

### Low-value care recommendations and measures by function in health care

**Fig. 2:**
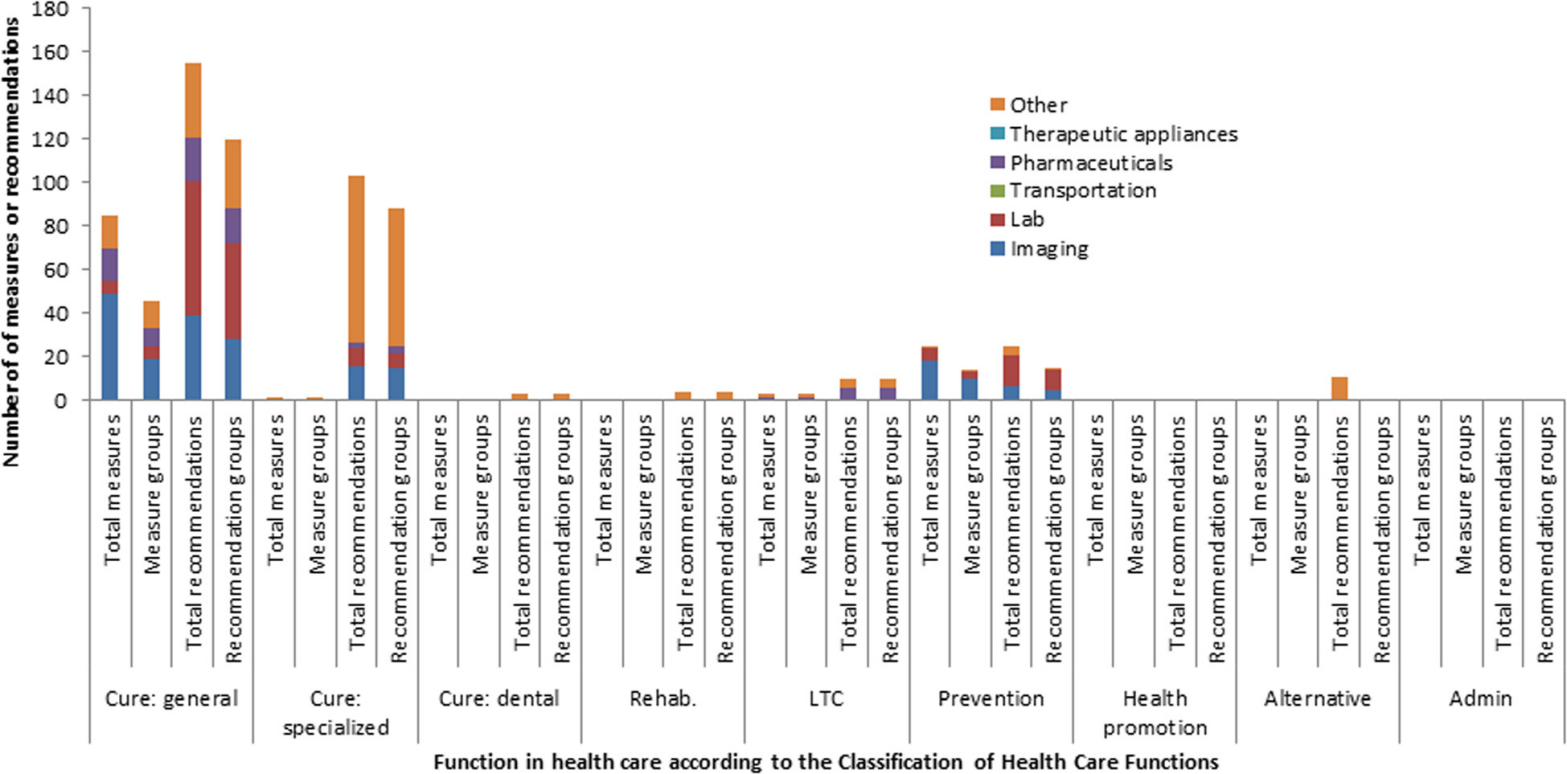
Number of low-value care recommendations and measures categorized by the OECD/WHO/Eurostat Classification of Health Care Functions (*n =* 426)*. Admin.: Administrative; Alternative: Traditional, Complementary and Alternative Medicine; LTC: Long Term Care; Rehab.: Rehabilitative care; *We yielded 115 low-value care measures and 412 recommendations from the literature. Since 101 recommendations had a similar subjects as the measures, we subtracted these from 412 recommendations. That leaves 311 recommendations. Therefore, the total recommendations and measures in figure is 311 + 101 = 426

Figure 2 displays an overview of low-value care recommendations and measures categorized by health care function [23]. Here, we combined recommendations and measures covering the same combination of diagnosis and procedure. For instance, we found 8 measures for imaging in low back pain (measure no. 2–9, Additional file 2) using slightly varying exclusion criteria regarding e.g. age category (18–50 years versus 18–55 years) or intervention (imaging in general versus specific MRI). These eight measures were combined into a single group. In this manner, we found that 115 measures and 101 low-value care recommendations corresponded with 65 measure groups. The remaining recommendations (*n =* 412-101 = 311) were aggregated into 241 new recommendation groups.

In the cure dimension we found 87 measures, which we further subdivided in general care (*n =* 85) and specialized care (*n =* 2). Most measures in the cure dimension were in imaging (*n =* 50) or pharmaceutical goods (*n =* 15). The remaining measures were categorized in long-term care (*n =* 3) and secondary prevention (*n =* 25).

### Quality of low-value care measures

#### Development process

Approximately half of the measures (*n =* 62) originated from low-value care recommendations (group A). Although the authors of the articles [2, 8, 11, 19, 26] described the methods to operationalize the low-value care measures, it was not specifically described how each specific low-value care recommendation was translated into a measure, i.e. how the denominator, numerator, exclusion and direction were determined for the purpose of the study. We did find that the measures developed by clinicians (*n =* 18) [8, 19] used (a combination of) International Classification of Diseases (ICD-9) and/or current procedural terminology (CPT) codes to construct the denominator [8, 19, 26].

The other half of the measures (*n =* 54) were developed by institutions (group B), including the NQF (*n =* 25) [8, 18, 19], the AHRQ (*n =* 10) [18, 19, 26], CMS QualityNet (*n =* 16) [10, 18, 19] and Blue Cross Blue Shield (*n =* 2) [19].

#### Level of evidence

Table 2 shows the level of evidence provided in the referenced sources for each measure. In group A, the recommendations were mainly derived from CW, USPSTF, and NICE (*n =* 45). Other group A measures originated from guidelines, peer-reviewed literature or sources that summarized low-value services [27].

**Table 2:** Level of evidence of low-value care measures

Level of evidence	Group A: Recommendation source	Measure numbers^a^	Count
1	CW, NICE or USPSTF recommendations; Guideline; Literature evidence (review or clinical trial)	39, 40, 46	3
2	CW, NICE or USPSTF recommendations; Literature evidence (review or clinical trial)	13, 14, 19, 20, 22, 23, 24, 25, 26, 44, 48, 50, 55, 77, 80, 90, 95, 103, 112, 115	20
2	CW, NICE or USPSTF recommendations; Guideline	33, 53	2
2	Literature evidence (reviews or clinical trial)	3, 21, 58, 76, 78, 81, 82, 83, 85, 89	10
2	Guideline	54, 57	2
3	USPSTF concludes that evidence is insufficient	101	1
Unknown	Literature: other compiled low-value service lists	47, 49, 113	3
Unknown	USPSTF recommendation not found	104, 107	2
Unknown	CW, NICE or USPSTF recommendations	34, 38, 43, 45, 51, 52, 59, 61, 84, 92, 98, 102, 105, 106, 108, 109, 110, 111, 114	19
Level of evidence	Group B: Institutional measure status	Measure numbers^a^	
1	NQF endorsed	5, 11, 16, 18, 41, 56, 62–67, 72, 73, 91, 93, 94, 96, 97	19
2	AHRQ measure supported by a clinical practice guideline or other peer-reviewed synthesis of clinical research evidence and one or more research studies published in a National Library of Medicine (NLM) indexed, peer-reviewed journal	60, 69, 70	3
2	AHRQ measure supported by a clinical practice guideline or other peer-reviewed synthesis of the clinical research evidence	1, 4, 55, 86	4
2	CMS QualityNet	2, 7, 8, 9, 27, 28, 29, 30, 31, 32, 37, 42, 99, 100	14
3	NQF endorsement removed since April 2014	6, 10, 74, 75	4
Unknown	NQF endorsement not found	17, 71	2
Unknown	AHRQ measure/guideline not found	68, 79, 87	3
Unknown	CMS QualityNet under revision	15	1
Unknown	CMS not found	12	1
Unknown	BCBS AQC measures not found	35, 36	2

*AHRQ* Agency for Healthcare Research and Quality, *BCBS AQC* Blue Cross Blue Shield, The Alternative Quality Contract, *CMS* Centers for Medicare & Medicaid Services, *CW* Choosing Wisely, *IOM* Institute of Medicine, *NICE* National Institute for Clinical Excellence (UK): do not do recommendations, *NQF* National Quality Forum, *USPSTF* United States Preventive Services Task Force

^a^: measure numbers are in correspondence with Additional file 2

Three measures (measure no. 39, 40, 46; Additional file 2) were assigned the highest level of evidence (1), as they were underpinned by guidelines and literature (trial or review) and recommendations. For most measures (*n =* 33), however, we found guideline or literature evidence solely. For one measure (measure no. 101) the USPSTF considered the evidence for the underlying recommendation insufficient to assess the benefits and harms of the procedure, which we therefore assigned with the lowest level of evidence. At the time of our review, for 24 measures, we considered the level of evidence to be unknown.

In group B, we found 19 measures [8, 18] supported by a quality label (NQF). For six measures the NQF endorsement was removed (*n =* 4) or not found (*n =* 2). Although the AHRQ website provides detailed information on the measures, we found no quality label, such as the NQF endorsement. We found seven measures (measure no. 1, 4, 55, 60, 69, 70, 86) displaying measurement characteristics (e.g. domain (process/outcome), description of denominator and numerator and target population) and evidence supporting the measure. The measures derived from QualityNet [28] were described in detail, however, no evidence supporting the description was provided.

#### Validity

Table 3 shows the validity of the five measures that were found most frequently (*n =* 26). Two measures had the highest level of evidence. Our search yielded no information on coding/criterion validity and construct validity for the included measures, while four out of five measures are currently used in practice.

**Table 3:** Validity of the top five published low-value care measures

	Preoperative cardiac tests for non-cardiac low-risk surgery	Antibiotics for upper respiratory tract infections	Imaging for low-back pain	Cervical cancer screening	Imaging for sinusitis diagnosis
Number of measures included in review ^a^	4 (measure no.: 42–44, 48)	7 (measure no.: 57–59, 62, 63, 65, 66)	8 (measure no.: 2–9)	3 (measure no.: 110–112)	4 (measure no.: 33, 35, 36, 59)
Measure criteria ^b^					
Face validity ^c^	Yes: level of evidence is 2	Yes, level of evidence is 1	Yes, level of evidence is 1	Yes, level of evidence is 2	Yes, level of evidence is 2
Coding/criterion validity	Not found	Not found	Not found	Not found	Not found
Construct validity	Not found	Not found	Not found	Not found	Not found
Used in practice	Yes, for payment determination (Hospital Outpatient Quality Reporting) [49]	Yes, in Physician Quality Reporting System [49]	Yes, for payment determination (Hospital Outpatient Quality Reporting) [49]	Yes, in Physician Quality Reporting System [49]	Not found

^a^: Measure numbers corresponding with Additional file 2 between brackets

^b^: Criteria for quality measures (AHRQ)

^c^: For level of evidence also see Table 2

## Discussion

To the best of our knowledge, this is the first systematic literature review identifying, categorizing and assessing the scope and quality of low-value care measures. We obtained 115 low-value care measures from the literature. Out of these 115 measures, 87 focused on the cure sector (primary and specialized care), 25 on secondary prevention and 3 on long-term care. Most measures (*n =* 62) originated from low-value care recommendations, while 53 were previously developed by institutions as the National Quality Forum. Three measures were assigned the highest level of evidence, as they were underpinned by both guidelines and literature evidence. For other measures, such a level of evidence was not transparently apparent. We do not conclude that these measures are invalid, because validity tests may not have been performed at all. Nevertheless, a lack of evidence is present at least. Our search yielded no information on coding/criterion validity and construct validity for the included subset of measures in this emerging field. Despite this, most measures are currently used in practice.

Low-value care measures have received increased attention and are now used for monitoring purposes, alignment of financial incentives [13, 29] and, in the foreseeable future, in shared saving programs such as the Alternative Quality Contract (AQC) [30]. In this manner, low-value care measurement may incentivize providers and insurers to shift resources from low-value services to high-value services [31]. Our findings show that more attention is needed for the evidential underpinning and quality of these measures. Otherwise, the lack of transparency and evidence will reduce acceptance of low-value care measures by its users. Additionally, using measures of low quality, might lead to negative consequences including underuse of indicated services, cost-shifting, damages to the patient-physician relationship, provider dissatisfaction, adverse health effects, or patient selection [17].

Our review showed that more than half of the low-value care measures originated from low-value service recommendations (i.e. CW, NICE, USPSTF). This implies that the empirical evidence of many low-value care measures is based on the evidence supporting the underlying low-value service recommendations. However, criteria for the development of recommendation lists remains rather vague in the CW initiative, as well as in other similar campaigns [7]. Therefore, more transparency regarding the evidential underpinning of the recommendations is needed. Next to the importance of evidence underlying both low-value service recommendations and measures, one should be aware that the aim of low-value service recommendations differs from the aim of low-value care measures. The aim of CW recommendations is patient and physician awareness, while the aim of low-value care measures in turn may be to inform decisions on several levels. Consequently, requirements for the quality and development of recommendations and measures approaches vary accordingly.

We found that most current low-value care measures are concentrated in the cure sector even though it was argued that low-value services are provided and used along the entire continuum of care [21]. For example, we only found four low-value care recommendations (that could possibly be transformed into low-value care measures) in rehabilitative care and none in the health promotion domain. This is probably the result of most measures originating from the CW initiative, which has its origin in the cure sector. While we acknowledge the emerging state of the field of research, we emphasize that similar consensus-based efforts are needed to stimulate the development of measures in other settings to broaden the scope and impact of the low-value care concept.

Given the potential impact of using low-value care measures, it is essential that guidelines for developing them be created by combined efforts of the involved parties: physicians, citizens, government and insurers [17, 32]. We do not suggest creating an evidence base for *each* health care intervention demonstrating all circumstances in which it is *not* effective. This will prove an undoable exercise. Expert judgement by the clinician will always remain necessary to some degree. Therefore, other types of information, e.g. from studies on practice variation in procedure rates or cost-effectiveness studies, will remain necessary to identify inefficiencies in healthcare, especially when high quality low-value care measures are not available. We do propose using expert opinion from initiatives such as Choosing Wisely as a starting point for monitoring low-value care. These qualitative information sources can be complemented with new scientific insights. For example, the insight that certain genes predict the development of breast cancer, must be used to prevent a considerable amount of low-value care utilization. Still, as soon as we start measuring and monitoring low-value care in such areas, it will be of particular interest to fully specify and define all measurement information, such as exclusion criteria, direction and evidence supporting the measure, and to make this publicly available. Furthermore, low-value care measures should be extensively tested regarding their level of evidence and validity before implementing them for use in practice, and specifically for the measures that are already in use. Recently, articles started studying aspects that are closely related to validity. As for example, Schwartz et al. [2] who found that the sensitivity and specificity strongly depends on the definition of the measures. Notwithstanding the efforts already been made, we stress the importance of the validity of the measures specifically being studied. Another area of research would be to further standardize low-value care measures, which ideally would result in alignment of the low-value care metrics and determining specifically for what subgroup or population a service is of low-value [2, 33]. Moreover, the guidelines should take into account any differences between countries in terms of the availability and provision of healthcare services that are likely to occur due to cultural or economic differences.

Another important issue to pay further attention to is the data requirements. Measuring low-value care utilization requires information on services provided to patients in combination with diagnosis and possibly additional patient characteristics. It is not clear to which extent current data sources can provide this information [2, 3], since rather detailed data need to be registered and data sources, such as claims data and detailed (hospital) registration data need to be connected in order to retrieve the necessary information.

### Limitations

Our study has two main limitations. First, we did not evaluate the quality of each individual measure. Ideally, we would extensively assess each measure regarding their validity. To perform this task for 115 measures was, however, beyond the scope of this review. Nonetheless, we performed a first attempt in assessing the validity for the five measures that appeared most often in the literature and highlight several important general quality issues. Second, we did not include grey literature in our search. Therefore, we may have missed relevant measures. Nevertheless, for the purpose of our review, namely to systematically map the state of affairs of low-value care measurement, we are confident that the publications we did use provided sufficient evidence.

## Conclusions

To conclude, our systematic review provides insight in the current state of low-value care measures. It shows that current low-value care measures only cover a selective part of the health care system. To achieve their full potential, future research should be focused on generating clear information about the level of evidence and validity to identify measures that truly represent low-value care in this emerging field of research. This will contribute to creating and maintaining the support of stakeholders who will use these measures for monitoring purposes and innovative insurer-provider contracts, all aiming to improve efficiency in health care with better health outcomes.

## Additional files

Additional file 1: Search strategy. (DOCX 16 kb) Additional file 2: Low-value care measures including numerator, denominator, exclusion criteria, direction and measure source and reference specified by function according to the OECD/WHO/Eurostat Classification of Health Care Functions (*n =* 115). (DOCX 182 kb) Additional file 3: Low-value care recommendations. (DOCX 78 kb)

## Abbreviations

AHRQ: Agency of Healthcare Research and Quality
AQC: Alternative quality contract
CMS: Centers of Medicare and Medicaid Services
CPT: Current procedural terminology
CW: Choosing wisely
e.g.: exempli gratia
i.e.: id est
ICD: International Classification of Diseases
ICHA-HC: Classification of health care functions
NICE: National Institute of Clinical Excellence
NQF: National Quality Forum
OECD: Organization for Economic Co-operation and Development
U.S.: United States
USPSTF: United States Preventive Services Task Force
WHO: World Health Organization
