# Supplementary material for: Are low-value care measures up to the task? A systematic review of the literature
Source: BMC Health Serv Res. 2016 Aug 18;16:405. doi: 10.1186/s12913-016-1656-3 (PMC4990838; doi:10.1186/s12913-016-1656-3)
Supplement: Additional file 1: — Search strategy. (DOCX 16 kb) [file 12913_2016_1656_MOESM1_ESM.docx]

**Additional file 1. Search strategy**

Database: MEDLINE 1950 to present, MEDLINE In-Process & Other Non-Indexed Citations

Search Strategy:

--------------------------------------------------------------------------------

1 *health services/ or *adolescent health services/ or *community health services/ or *child health services/ or *community health nursing/ or *community mental health services/ or *community pharmacy services/ or *community health centers/ or *home care services/ or *maternal health services/ or *occupational health services/ or *preventive health services/ or *preventive medicine/ or *dental health services/ or *emergency medical services/ or *emergency service, hospital/ or *hospitals/ or *health services for the aged/ or *mental health services/ or *nursing services/ or *personal health services/ or *pharmaceutical services/ or *rehabilitation/ or *reproductive health services/ or *rural health services/ or *suburban health services/ or *women's health services/ or *mass screening/ (295687)

2 *primary health care/ or *general practice/ or *family practice/ or *physicians, family/ or *professional practice/ or *physicians practice patterns/ or *comprehensive health care/ or *managed care programs/ or *delivery of health care/ or *delivery of health care, integrated/ or *patient care management/ or *nursing process/ or *nursing/ or *nurse practitioners/ or *telemedicine/ or *health services administration/ or *health services research/ or *translational medical research/ or *health facility administration/ or *health facilities/ or *health maintenance organizations/ or *health planning/ or *regional health planning/ or *community health planning/ or *regional medical programs/ or *health policy/ or *national health programs/ or *social work/ or *social welfare/ or *child welfare/ or *infant welfare/ or *maternal welfare/ or *government regulation/ or *government programs/ or *multi-institutional systems/ (344106)

3 (health system* or healthcare or health service* or care or (health and services) or health planning or health policy or health reform or welfare or preventive service* or screening).ti. (523842)

4 (family practic* or family physician* or general practi* or "gps" or "gp’s" or nurse practitioners or community health center* or community health centre* or municipal health center* or municipal health centre* or health center* or health centre*).ti. (47193)

5 "quality of health care"/ or "quality indicators, health care"/ or health care costs/ (93908)

6 1 or 2 or 3 or 4 or 5 (1008916)

7 ((identif* or indicator* or correlat* or pattern* or predictor* or measur* or assess* or examin* or classify* or categoriz* or characterization or quantif* or variation*) adj5 (low value or "no value" or overuse or overutilization or overused or misuse or disuse or (wasteful adj3 "use") or (wasteful adj3 services))).tw. (1541)

8 ((estimat* or evaluat* or distinguishing or labe?ling or compar* or potential* or factors or prevalence or rate* or degree or evidence or understanding) adj5 (low value or "no value" or overuse or overutilization or overused or misuse or disuse or (wasteful adj3 "use") or (wasteful adj3 services))).tw. (2408)

9 ((identif* or indicator* or correlate* or pattern* or predictor* or measur* or assess* or examin* or classify* or categoriz* or characterization or quantif* or variation*) adj5 ((unsafe adj3 practice*) or (ineffective adj3 practice*) or (ineffective adj3 care) or (ineffective adj3 healthcare) or (inappropriat* adj3 practice*) or (inappropriat* adj3 "use") or (harmful adj3 practice*))).tw. (490)

10 ((estimat* or evaluat* or distinguishing or labe?ling or compar* or potential* or factors or prevalence or rate* or degree or evidence or understanding) adj5 ((unsafe adj3 practice*) or (ineffective adj3 practice*) or (ineffective adj3 care) or (ineffective adj3 healthcare) or (inappropriat* adj3 practice*) or (inappropriat* adj3 "use") or (harmful adj3 practice*))).tw. (810)

11 ((identif* or indicator* or correlate* or pattern* or predictor* or measur* or assess* or examin* or classify* or categoriz* or characterization or quantif* or variation) adj5 waste).tw. (2127)

12 ((estimat* or evaluat* or distinguishing or labe?ling or compar* or potential* or factors or prevalence or rate or degree or evidence or understanding) adj5 waste).tw. (2833)

13 ((index or instrument) and (low value or "no value" or overuse or overutilization or overused or misuse or disuse or (wasteful adj3 "use") or (wasteful adj3 services))).ti. (21)

14 ("choosing wisely" or "do not do recommendations").tw. (134)

15 6 and (7 or 8 or 9 or 10 or 11 or 12 or 13 or 14) (1126)

16 prescription drug misuse/ or (prescription misuse or substance misuse or drug misuse or alcohol or hazardous drinking or substance).ti. (100941)

17 (hospital waste or medical waste or biomedical waste or bio-medical waste or healthcare waste or health-care waste or care waste or genotoxic waste or plate waste or waste anesthetic gases or infectious waste or pharmaceutical waste or solid waste or liquid waste or aeration or waste disposal or waste treatment or electronic waste or waste waster* or waste materials or aeration).ti. (4893)

18 15 not (16 or 17) (927)

19 (practice* or low value or value or "no value" or overuse or misuse or overutilization or overused or inappropr* or ineffective or unsafe or "use" or waste).ti. or ("choosing wisely" or "do not do recommendations").tw. (664168)

20 18 and 19 (487)

21 20 and english.lg. (452)

22 limit 21 to yr=2010-2015 (227)

23 22 not (letter or comment or news).pt. (205)

24 remove duplicates from 23 (201)
