# Supplementary material for: Are low-value care measures up to the task? A systematic review of the literature
Source: BMC Health Serv Res. 2016 Aug 18;16:405. doi: 10.1186/s12913-016-1656-3 (PMC4990838; doi:10.1186/s12913-016-1656-3)
Supplement: Additional file 2: — Low-value care measures including numerator, denominator, exclusion criteria, direction and measure source and reference specified by function according to the OECD/WHO/Eurostat Classification of Health Care Functions (n = 115). (DOCX 182 kb) [file 12913_2016_1656_MOESM2_ESM.docx]

Additional file 2. Low-value care measures including numerator, denominator, exclusion criteria, direction and measure source and reference specified by function according to the OECD/WHO/Eurostat Classification of Health Care Functions (n=115)

| **No.** | **Measure** | **Measure details** | | | | **Original source** | | **Ref.** | **Function** | | **Non-function** |
| --- | --- | --- | --- | --- | --- | --- | --- | --- | --- | --- | --- |
|  |  | **Numerator*** | **Denominator*** | **Exclusion** | **Direction** | **Measure** | **Recommendation** |  |  |  |  |
| 1 | Barium swallow test for GERD | % patients ≥ 18 seen for an initial evaluation of GERD who did not have a barium swallow test ordered | | - | higher rate is better performance | NGC | - | Chan [^19^](#_ENREF_19) | Cure | General | Imaging |
| 2 | MRI Back | All patients undergoing an MRI of the lumbar spine with a diagnosis of low back pain without evidence of antecedent conservative therapy (e.g. physical therapy within 60 d of the MRI, chiropractic manipulation within 60 d of the MRI, or low back pain evaluation and management using CPT codes within 28 to 60 d of the MRI-during which time the patient is likely to have been educated or home exercises are likely to have been recommended). | All patients undergoing an MRI of the lumbar spine with a diagnosis of low back pain. | MRI's performed in patients with diagnosis of cancer, trauma, intravenous drug abuse, neurological impairment, immune deficiency, or intra spinal abscess. | - | CMS / QualityNet | - | Mathias [^12^](#_ENREF_12) | Cure | General | Imaging |
| 3 | Back pain images for patients with nonspecific low back pain | Back imaging with a diagnosis of lower back pain | Patients with back pain | - | - | - | CW/ NICE / Lit | Schwartz [^3^](#_ENREF_3) | Cure | General | Imaging |
| 4 | X-ray for back pain in adults aged 18-55 y | Visits by adults with acute back pain who received x-ray | Visits by adults with acute back pain | Visits by adults with malignancy, weight loss, fever, cachexia, or neurologic signs | - | NCQA | - | Kale [^25^](#_ENREF_25) | Cure | General | Imaging |
| 5 | Imaging for low back pain | % members aged 18-50 with negative diagnosis history who had outpatient or ER visit with primary diagnosis of low back pain who did not have an imaging study (plain x-ray, MRI CT scan) within 28 days of this visit. Members with any low back pain diagnosis during the 180 days of the index visit, who have a diagnosis of cancer, who had a diagnosis in the prior 12 months of recent trauma, intravenous drug abuse, r neurological impairment are excluded. | | - | higher rates is better performance | NGC / NQF | - | Chan [^19^](#_ENREF_19) | Cure | General | Imaging |
| 6 | Imaging for acute back pain | % patients with a diagnosis for back pain for whom the physician ordered imaging studies during the 6 weeks after pain onset, in the absence of 'red flags'. | | - | lower rates is better performance | NQF | - | Chan [^19^](#_ENREF_19) | Cure | General | Imaging |
| 7 | MRI lumbar spine for low back pain | % MRI of the lumbar spine studies with a diagnosis of low back pain on the imaging claim and for which the patient did not have prior claims-based evidence of antecedent conservative therapy | | - | lower rates is better performance | QualityNet | - | Chan [^19^](#_ENREF_19) | Cure | General | Imaging |
| 8 | MRI lumbar spine for low back pain | MRI of the lumbar spine studies with a diagnosis of low back pain (from the denominator) without the patient having claims-based evidence of prior antecedent conservative therapy. CPT=72148, or 72149, or 72158 with no codes for 97110, 97112, 97113, 97124, 97140, 98940, 98941, 98942, 98943 in the 60 days preceding the MRI of the lumbar spine AND no codes for 99210-99205, 99211 -99215, 99241-99245, 99341-99345, 99347-99350, 99354-99357, 99385-99387, 99395-99397, 99401-99404, 99455-99456, 99499 between 28 and 60 days preceding the MRI of the lumbar spine | MRI of the lumbar spine studies with a diagnosis of low back pain on the imaging claim. CPT=72148, or 72149, or 72158 AND ICD-9: 721.3, 721.90, 722.10, 722.52, 722.6, 722.93, 724.02, 724.2, 724.3, 724.5, 724.6, 724.70, 724.71, 724.79, 738.5, 739.3, 739.4, 846.0, 846.1, 846.2, 846.3, 846.8, 846.9 , 847.2 | Excluded from the denominator - CPT codes: 22010-22865 and 22899 in 90 days preceding MRI; ICD-9 codes: 140-208, 230-234, 235-239, 304.0X, 304.1X, 304.2X, 304.4X, 305.4X, 305.5X, 305.6X, 305.7X, 344.60, 344.61, 729.2, 042-044, 279.3 in preceding 365 days; 800-839, 850-854, 860-869, 905-909, 926.11, 926.12, 929, 952, 958-959 in preceding 45 days; 324.9, 324.1 on same claim as MRI | - | QualityNet | - | Segal [^18^](#_ENREF_18) | Cure | General | Imaging |
| 9 | Don’t do imaging for low back pain when no red flags are present | Beneficiaries who received a low back x-ray, CT or MRI within six weeks of incident low back pain diagnosis | Beneficiaries with low back pain over age 65 without other imaging indication | Prior diagnosis of low back pain, trauma and neurological impairment, within previous 12 months and cancer at any point during study period: E code (external causes of injury) or trauma diagnosis on imaging event claim | - | NQF | CW | Colla [^9^](#_ENREF_9) | Cure | General | Imaging |
| 10 | Repeat imaging studies for back pain | % patients who received inappropriate repeat imaging studies in the absence of red flags or progressive symptoms | | - | lower rates is better performance | NQF | - | Chan [^19^](#_ENREF_19) | Cure | General | Imaging |
| 11 | Cardiac stress imaging (routine testing after percutaneous coronary intervention, PCI) | % of all stress SPECT MPI, stress echo, CCTA, and CMR performed routinely after PCI, with reference to timing of test after PCI and symptom status. | | - | lower rates is better performance | NQF | - | Chan [^19^](#_ENREF_19) | Cure | General | Imaging |
| 12 | Overuse of stress testing | % patients ≥ 18 with a CAD diagnosis for ≥ 12 months but no documentation of AMI OR unstable angina OR referral for cardiac rehabilitation during measurement period who received ≥ 2 stress tests within a 12-month period. | | - | lower rates is better performance | CMS | - | Chan [^19^](#_ENREF_19) | Cure | General | Imaging |
| 13 | Stress echocardiography in symptomatic or ischemic equivalent acute chest pain | Individuals with CPT codes as listed or HCPCS codes as listed for echocardiography | Individuals with a code for emergency visit* with any of the ICD-9 diagnoses OR individuals with a hospitalization with DRGs as listed, or primary or secondary diagnosis code during hospitalization for any of the ICD-9 diagnoses | - | - | - | CW / Lit | Segal [^18^](#_ENREF_18) | Cure | General | Imaging |
| 14 | Stress testing for stable coronary disease | Stress testing for patients with an established diagnosis of ischemic heart disease or angina (≥6 months before the stress test) and thus not done for screening purposes | IHD patients | Test not associated with inpatient or emergency care, which might be indicative of unstable angina; only patients with a past diagnosis of myocardial infarction to exclude patients with a history of non-cardiac chest pain inaccurately coded as angina (i.e., those with no underlying ischemic heart disease who might benefit from screening and optimization of medical management). | - | - | CW / Lit | Schwartz [^3^](#_ENREF_3) | Cure | General | Imaging |
| 15 | CT for headache in emergency department | OF ED visits with primary diagnosis of headache, the number with a brain CT on the same day; patients admitted to the hospital or have secondary diagnoses on claims indicating clinical need are excluded | | - | lower rates is better performance | CMS / Lewin Group | - | Chan [^19^](#_ENREF_19) | Cure | General | Imaging |
| 16 | Head CT imaging for mild traumatic brain injury | % adult patients presenting within 24 hours of a non-penetrating head injury with a Glasgow coma score >13 and underwent head CT for trauma in the ED who have a documented evidence-based indication prior to imaging | | - | higher rates is better performance | NQF | - | Chan [^19^](#_ENREF_19) | Cure | General | Imaging |
| 17 | MRI in adults with mild traumatic brain injury | MRI on the same claim as diagnosis if outpatient or during hospitalization if inpatient | Patients with traumatic brain injury | - | - | NQF | - | Segal [^18^](#_ENREF_18) | Cure | General | Imaging |
| 18 | Cervical spine imaging in trauma | % adult patients undergoing cervical spine radiographs or CT for trauma who fulfill NEXUS low-risk criteria for cervical spine injury or Canadian Cervical Spine Rule documented prior to imaging. Patients are excluded if they have communication difficulties, inadequate prior radiographs, or for whom further imaging is indicated. | | - | higher rates is better performance | NQF | - | Chan [^19^](#_ENREF_19) | Cure | General | Imaging |
| 19 | EEG for headaches | EEG with headache diagnosis in the claim (specified with claim codes) | Patients with headache diagnosis | - | - | - | CW / Lit | Schwartz [^3^](#_ENREF_3) | Cure | General | Imaging |
| 20 | Head imaging for uncomplicated headache | CT or MR imaging of the head with a diagnosis of (non-thunderclap, non-posttraumatic) headache (specified with claim codes) | Patients with headache diagnosis | - | - | - | CW / Lit | Schwartz [^3^](#_ENREF_3) | Cure | General | Imaging |
| 21 | Bone mineral density testing at frequent intervals | Bone mineral density test less than 2 y after prior bone mineral density test (specified with claim codes) | Patients with osteoporosis | - | - | - | Lit | Schwartz [^3^](#_ENREF_3) | Cure | General | Imaging |
| 22 | Don’t routinely repeat dual-energy x-ray absorptiometry (DXA) scans more often than once every two years | DXA scans performed on female beneficiaries at low risk for fracture within 23 months of a previous scan | DXA scans performed on female beneficiaries over age 66 at low risk for fracture | Fragility fracture or cancer diagnosis within 23 months of the index DXA scan | - | - | CW | Colla [^9^](#_ENREF_9) | Cure | General | Imaging |
| 23 | Short interval dual energy x-ray absorptiometry scans (DXAs): population rate | DXA scans performed on female beneficiaries over age 66 at low risk for fracture | Number of DXAs performed per 100 female beneficiaries over age 66 | We excluded beneficiaries not continuously enrolled in fee-for-service Medicare Parts A and B in the 23 months prior to each DXA test identified. We conservatively excluded beneficiaries who were (i) diagnosed with any cancer, except non-melanoma skin cancer (using the Clinical Classifications [17] or (ii) diagnosed with fragility fracture in the 23 months. | - | - | CW | Morden [^13^](#_ENREF_13) | Cure | General | Imaging |
| 24 | Short interval dual energy x-ray absorptiometry scans (DXAs): short interval rate | DXA scans performed on female beneficiaries over age 66 at low risk for fracture | Number of DXAs performed per within 23 months of a previous DXA per 100 female beneficiaries aged over 66 | We excluded beneficiaries not continuously enrolled in fee-for-service Medicare Parts A and B in the 23 months prior to each DXA test identified. We conservatively excluded beneficiaries who were (i) diagnosed with any cancer, except non-melanoma skin cancer (using the Clinical Classifications [17] or (ii) diagnosed with fragility fracture in the 23 months. | - | - | CW | Morden [^13^](#_ENREF_13) | Cure | General | Imaging |
| 25 | Short interval dual energy x-ray absorptiometry scans (DXAs): proportion of inappropriate DXAs | DXA scans performed on female beneficiaries over age 66 at low risk for fracture | The proportion of all DXAs done at a inappropriately short interval (occurring within 23 months of a previous DXA) | We excluded beneficiaries not continuously enrolled in fee-for-service Medicare Parts A and B in the 23 months prior to each DXA test identified. We conservatively excluded beneficiaries who were (i) diagnosed with any cancer, except non-melanoma skin cancer (using the Clinical Classifications [17] or (ii) diagnosed with fragility fracture in the 23 months. | - | - | CW | Morden [^13^](#_ENREF_13) | Cure | General | Imaging |
| 26 | Short interval dual energy x-ray absorptiometry scans (DXAs): mean intertest time interval | DXA scans performed on female beneficiaries over age 66 at low risk for fracture | The mean inter-test time interval for DXAs done within 23 months of a previous DXA. | We excluded beneficiaries not continuously enrolled in fee-for-service Medicare Parts A and B in the 23 months prior to each DXA test identified. We conservatively excluded beneficiaries who were (i) diagnosed with any cancer, except non-melanoma skin cancer (using the Clinical Classifications [17] or (ii) diagnosed with fragility fracture in the 23 months. | - | - | CW | Morden [^13^](#_ENREF_13) | Cure | General | Imaging |
| 27 | Abdomen CT (use of contrast) | Of all abdomen CT studies performed (with contrast, without contrast, both with and without contrast-combined study), the number of abdomen CT combined studies (with and without contrast). Exclusions for specified diagnoses. | | - | unclear | QualityNet | - | Chan [^19^](#_ENREF_19) | Cure | General | Imaging |
| 28 | Abdomen CT use of contrast material | The number of Abdomen CT studies with and without contrast (“combined studies”). CPT 74170 | The number of Abdomen CT studies performed (with contrast, without contrast or both with and without contrast). CPT 74150, 74160, 74170 | - | - | QualityNet | - | Segal [^18^](#_ENREF_18) | Cure | General | Imaging |
| 29 | CT Abdomen | All combined abdominal CT's. | Total number of abdominal CTs performed with, without, or with and without contrast. | CTs performed in patients with diagnoses of hematuria, pancreatic disorders, adrenal masses, unspecified disorders of the kidney and ureter, or malignant neoplasms of the liver, bile ducts, pancreas, kidney or liver. | - | CMS / QualityNet | - | Mathias [^12^](#_ENREF_12) | Cure | General | Imaging |
| 30 | CT Thorax | All combined thoracic CTs | Total number of thoracic CTs performed with, without, or with and without contrast. | No CTs. | - | CMS / QualityNet | - | Mathias [^12^](#_ENREF_12) | Cure | General | Imaging |
| 31 | Thorax CT (use of contrast) | Of all thorax CT studies performed (with contrast, without contrast, both with and without contrast-combined study), the number of thorax CT combined studies (with and without contrast). | | - | unclear | QualityNet | - | Chan [^19^](#_ENREF_19) | Cure | General | Imaging |
| 32 | Thorax CT use of contrast material | The number of thorax CT studies with and without contrast (“combined studies”). CPT 71270 | The number of thorax CT studies performed (with contrast, without contrast or both with and without contrast). CPT 71250, 71260, 71270 | Exclude from the denominator if on the same claim as CPT 74140, 74160, 74170 - ICD-9 code: 593.9, 120.0, 599.70, 599.71, 599.72, 251.2, 251.0, 250.8, 270.3, 255.9, 194.xx, 277.xx, 237.xx, 155.0, 155.1, 155.2, 157.0, 157.1,157.2,157.3, 157.4, 157.8, 157.9, 189.0, 211.5, 211.6, 211.7, 223.0 | - | QualityNet | - | Segal [^18^](#_ENREF_18) | Cure | General | Imaging |
| 33 | CT of the sinuses for uncomplicated acute rhinosinusitis | Maxillofacial CT study with a diagnosis of sinusitis in the imaging claim (specified with claim codes) | Patients with sinusitis diagnosis | - | - | - | CW / Lit | Schwartz [^3^](#_ENREF_3) | Cure | General | Imaging |
| 34 | Don’t order upper-tract imaging for patients with benign prostatic hyperplasia (BPH) | Beneficiaries who received an intravenous pyelogram or an abdominal CT, MRI, or ultrasound within 60 days of the index diagnosis | Male beneficiaries diagnosed with BPH over age 65 without other indications of imaging | Cancer diagnosis at any point during study period (e.g. chronic renal failure, nephritis, calculus of kidney and ureter, kidney stones abdominal pain) within 60 days of diagnosis | - | - | CW | Colla [^9^](#_ENREF_9) | Cure | General | Imaging |
| 35 | Fiberoptic laryngoscopy for sinusitis diagnosis | Laryngoscopy WITH ICD-9 code indicating sinusitis on the same claim | Individuals with a diagnosis of sinusitis (acute or chronic) –inpatient or outpatient | - | - | AQC | - | Segal [^18^](#_ENREF_18) | Cure | General | Imaging |
| 36 | Nasal endoscopy for sinusitis diagnosis | Nasal endoscopy WITH ICD-9 code indicating sinusitis on the same claim | Individual with a diagnosis of sinusitis (acute or chronic) –inpatient or outpatient | - | - | AQC | - | Segal [^18^](#_ENREF_18) | Cure | General | Imaging |
| 37 | Simultaneous use of brain CT and sinus CT | % brain CT with a sinus CT performed on the same day at the same facility. Exclusions for specified diagnoses noted in one of the diagnoses fields of the brain CT claim. | | - | lower rates is better performance | QualityNet | - | Chan [^19^](#_ENREF_19) | Cure | General | Imaging |
| 38 | EEG monitoring in individuals presenting with syncope | EEG on the same claim as diagnosis of syncope or at any time during the hospitalization with a code for syncope | Individuals with an outpatient visit with diagnosis of syncope or hospitalization for syncope | - | - | - | NICE | Segal [^18^](#_ENREF_18) | Cure | General | Imaging |
| 39 | Head imaging in the evaluation of syncope | CT or MR imaging of the head with a diagnosis of syncope in the imaging claim (specified with claim codes) | Patients with syncope diagnosis | - | - | - | CW / NICE | Schwartz [^3^](#_ENREF_3) | Cure | General | Imaging |
| 40 | Screening for carotid artery disease for syncope | Carotid imaging with syncope diagnosis (specified with claim codes) | Patients with syncope diagnosis | - | - | - | CW / NICE | Schwartz [^3^](#_ENREF_3) | Cure | General | Imaging |
| 41 | Imaging for patients at low risk for pulmonary embolism (PE) | Number of hemodynamically stable patients who receive CT pulmonary angiograms for suspected PE who have either: (a) a low clinical probability of PE (determined by structured prediction tool or implicit judgment, prior to imaging), and a negative D-dimer OR (b) a low clinical probability of PE and no D-dimer performed OR (c) no pretest probability documented | | - | lower rates is better performance | NQF | - | Chan [^19^](#_ENREF_19) | Cure | General | Imaging |
| 42 | Cardiac imaging (preoperative risk assessment for non-cardiac low-risk surgery) | number of stress echocardiography, SPECT MPI and stress MRI studies performed at the hospital outpatients department within 30 days of an ambulatory low-risk non-cardiac surgery (e.g. endoscopic, superficial, cataract) performed at any location | | - | lower rates is better performance | QualityNet | - | Chan [^19^](#_ENREF_19) | Cure | General | Imaging |
| 43 | Don’t perform preoperative cardiac tests for low-risk, non-cardiac surgeries | Beneficiaries who received a non-indicated cardiac test, including stress tests, echocardiograms, electrocardiograms, CTs, MRIs or PETs within 30 days before low-risk surgery | Beneficiaries over age 65 undergoing low-risk, non-cardiac surgery (e.g. breast surgery, transurethral resection of the prostate, corneal transplant, inguinal hernia repair, lithotripsy, arthroscopy, laparoscopic cholecystectomy) | Appropriate clinical indication on testing event claim (e.g., palpitations) or admission in the 30 days before surgery | - | PQRS | CW | Colla [^9^](#_ENREF_9) | Cure | General | Imaging |
| 44 | Preoperative stress testing | Stress electrocardiography, echocardiography or nuclear medicine imaging specified as a preoperative assessment or occurring within 30 d before a low- or intermediate-risk non-cardiothoracic surgical procedure (specified with claim codes) | Patients undergoing selected surgeries | - | - | - | CW / Lit | Schwartz [^3^](#_ENREF_3) | Cure | General | Imaging |
| 45 | Don’t perform preoperative cardiac tests for cataract surgeries | Beneficiaries who received a non-indicated cardiac test, including stress tests, echocardiograms, electrocardiograms and advanced cardiac imaging in the 30 days before cataract surgery | Beneficiaries over age 65 undergoing cataract surgery | Appropriate clinical indication on testing event claim (e.g., palpitations) or admission in the 30 days before surgery | - | PQRS | CW | Colla [^9^](#_ENREF_9) | Cure | General | Imaging |
| 46 | Preoperative chest radiography | Chest radiograph specified as a preoperative assessment or occurring within 30 d before a low- or intermediate risk non-cardiothoracic surgical procedure (specified with claim codes) | Patients undergoing selected surgeries | - | - | - | CADTH / CW / Lit | Schwartz [^3^](#_ENREF_3) | Cure | General | Imaging |
| 47 | Preoperative chest radiography in the absence of a clinical suspicion for intrathoracic pathology | 71010, 71020 These codes must be in a 30 day window before the anesthesia code | All patients who had anesthesia 00100-02101 (CPT) | diagnoses 466.xx, 480.xx-488.xx, 490.xx-496.xx, 500.xx-508.xx, 510.xx-519.xx | - | - | Lit | Segal [^18^](#_ENREF_18) | Cure | General | Imaging |
| 48 | Preoperative echocardiography | Echocardiogram specified as a preoperative assessment or obtained within 30 days before a low- or intermediate-risk non-cardiothoracic surgical procedure (specified with claim codes) | Patients undergoing selected surgeries | - | - | - | CW / Lit | Schwartz [^3^](#_ENREF_3) | Cure | General | Imaging |
| 49 | Preoperative PFT | PFT specified as a preoperative assessment or occurring within 30 d before a low- or intermediate-risk surgical procedure (specified with claim codes) | Patients undergoing selected surgeries | - | - | - | CW | Schwartz [^3^](#_ENREF_3) | Cure | General | Imaging |
| 50 | PTH measurement for patients with stage 1-3 CKD | PTH measurement in patients with CKD (specified with claim codes) | CKD patients | - | - | - | NICE / Lit | Schwartz [^3^](#_ENREF_3) | Cure | General | Lab |
| 51 | Diagnostic tests, such as immunoglobulin testing, in the evaluation of allergy | Use of CPT 82701, 82784, 82785, 82787, 86005 on the same claim as a code for diagnoses in the denominator column | 477.0, 477.1, 477.2, 477.8, 477.9, 493.0, 493.02, 493.9, 493.90, 493.92, 708.0, 995.3 | - | - | - | CW | Segal [^18^](#_ENREF_18) | Cure | General | Lab |
| 52 | Serological tests for Helicobacter pylori | Any code indicating testing for H. pylori | Whole population | - | - | - | NICE | Segal [^18^](#_ENREF_18) | Cure | General | Lab |
| 53 | Hypercoagulability testing for patients with deep vein thrombosis | Laboratory tests for hypercoagulable states within 30 d after diagnosis of lower-extremity deep vein thrombosis or pulmonary embolism (specified with claim codes) | Patients with deep vein thrombosis | - | - | - | CW / Lit | Schwartz [^3^](#_ENREF_3) | Cure | General | Lab |
| 54 | Abx for acute asthma exacerbation | Visits by adults with acute asthma exacerbation who receive any abx | Visits by adults with acute asthma exacerbation | None | - | - | NAEPP | Kale [^25^](#_ENREF_25) | Cure | General | Pharmaceuticals |
| 55 | Abx for acute bronchitis | Visits by adults with bronchitis who received any abx | Visits by adults with acute bronchitis | Visits by adults with HIV, cystic fibrosis, cancer, chronic bronchitis, emphysema, bronchiectasis, extrinsic allergic alveolitis, chronic airway obstruction, tuberculosis, or pneumoconioses | - | NCQA | - | Kale [^25^](#_ENREF_25) | Cure | General | Pharmaceuticals |
| 56 | Antibiotics for acute bronchitis | % patients aged 18-64 with acute bronchitis who were not dispensed an antibiotic prescription | | - | higher rates is better performance | NGC / NQF | - | Chan [^19^](#_ENREF_19) | Cure | General | Pharmaceuticals |
| 57 | Abx for URTI | Visits by adults with uncomplicated URTI who received any antibiotic medication | Visits by adults with uncomplicated URTI | Visits by adults with HIV, COPD or cancer | - | - | ICSI | Kale [^25^](#_ENREF_25) | Cure | General | Pharmaceuticals |
| 58 | Abx other than nitrofurantoin, trimethoprimsulbactam, or quinolone use for UTI | Visits by female adults with uncomplicated UTI who received abx other than nitrofurantoin, trimethoprim-sulbactam, or quinolone | Visits by female adults with uncomplicated URTI | Visits by female adults with vaginitis/cervicitis, skin infections, kidney infections, STD, history of DM, cancer, pregnancy nephrolithiasis or urologic procedures | - | - | Lit | Kale [^25^](#_ENREF_25) | Cure | General | Pharmaceuticals |
| 59 | Sinus CT or antibiotics for uncomplicated acute rhinosinusitis | Any occurrence of sinus CT (CPT 70486, 70487, 70488) in the 3 months preceding the diagnosis of acute sinusitis | 461.0, 461.1, 461.2, 461.3, 461.8, 461.9 AND NO code in the preceding 3 months for any of these | code in the preceding 3 months for 473.0, 473.1, 473.2, 473.3, 473.8, 473. | - | - | CW | Segal [^18^](#_ENREF_18) | Cure | General | Pharmaceuticals |
| 60 | Medication use for urinary incontinence | % female patients ≥ 65 with urinary incontinence who were prescribed a medication to treat the urinary incontinence and who had a trial of behavioral therapy (e.g. bladder training, pelvic floor muscle training, prompted voiding) documented | | - | higher rates is better performance | NGC | - | Chan [^19^](#_ENREF_19) | Cure | General | Pharmaceuticals |
| 61 | Don’t use opioid or butalbital treatment for migraine, except as a last resort | Beneficiaries who filled an opioid or butalbital prescription within 21 days of the office visit with migraine diagnosis | Beneficiaries over age 65 with a diagnosed migraine and no other indication for opioids | An 'E' code, inpatient admission, back pain, abdominal pain, surgery, fracture, cancer or hospice enrollment within 60 days of index visit | - | - | CW | Colla [^9^](#_ENREF_9) | Cure | General | Pharmaceuticals |
| 62 | Antibiotics for pharyngitis without strep confirmation | % patients aged 2-18 diagnosed with pharyngistis who were dispensed an antibiotic and received Group A streptococcustest | | - | higher rates is better performance | NGC / NQF | - | Chan [^19^](#_ENREF_19) | Cure | General | Pharmaceuticals |
| 63 | Antibiotics for URI | % patients aged 3 months to 18 with URI who were not dispensed an antibiotic prescription | | - | higher rates is better performance | NGC / NQF | - | Chan [^19^](#_ENREF_19) | Cure | General | Pharmaceuticals |
| 64 | Antihistamines or decongestants for OME | % patients aged 2 months to 12 years with OME who were not prescribed or recommended to receive either antihistamines or decongestants except with documented medical rationale. | | - | higher rates is better performance | NGC / NQF | - | Chan [^19^](#_ENREF_19) | Cure | General | Pharmaceuticals |
| 65 | Systemic antimicrobial therapy for AOE | % patients aged ≥ 2 with AOE who were not prescribed systemic antimicrobial therapy except with documented medical rationale. | | - | higher rates is better performance | NGC / NQF | - | Chan [^19^](#_ENREF_19) | Cure | General | Pharmaceuticals |
| 66 | Systemic antimicrobial therapy for OME | % patients aged 2 months to 12 years with OME who were not prescribed systemic antimicrobials except with documented medical rationale. | | - | higher rates is better performance | NGC / NQF | - | Chan [^19^](#_ENREF_19) | Cure | General | Pharmaceuticals |
| 67 | Systemic corticosteroids for OME | % patients aged 2 months to 12 years with OME who were not prescribed systemic corticosteroid except with documented medical rationale. | | - | higher rates is better performance | NGC / NQF | - | Chan [^19^](#_ENREF_19) | Cure | General | Pharmaceuticals |
| 68 | Intravenous unfractionated heparin | % patients ≥ 18 with diagnosis of ischemic stroke who did not receive heparin. Patients undergoing carotid endarterectomy, carotid angioplasty-stenting, or had documented medical reasons for receiving heparin excluded | | - | - | NGC | - | Chan [^19^](#_ENREF_19) | Cure | General | Pharmaceuticals |
| 69 | Chronic wound care | % visits for patients ≥ 18 with a diagnosis of chronic skin ulcer without the use of a wound surface culture technique. | | - | higher rates is better performance | NGC | - | Chan [^19^](#_ENREF_19) | Cure | General | - |
| 70 | Chronic wound care | % visits for patients ≥ 18 with a diagnosis of chronic skin ulcer without a prescription or recommendation to use wet to dry dressings | | - | higher rates is better performance | NGC | - | Chan [^19^](#_ENREF_19) | Cure | General | - |
| 71 | More than 1 emergency department visit in last 30 days of life | More than 2 visits with location code or CPT code indicating ED use within 30 days before death | Individuals with death during our observation period | - | - | NQF | - | Segal [^18^](#_ENREF_18) | Cure | General | - |
| 72 | Caesarean section | Patients with ICD-9-CM Procedure Code for cesarean section (numerator); | nulliparous patients delivered of a live term singleton newborn in vertex presentation (denominator); | contraindications to vaginal delivery, age <8 or ≥ 65, length of stay >120 days, clinical trial enrollment (excluded) | lower rates is better performance | NQF | - | Chan [^19^](#_ENREF_19) | Cure | General | - |
| 73 | Elective delivery | % patients with elective vaginal deliveries or elective cesarean sections among all patients delivering newborns at ≥ 37 and < 39 weeks of gestation completed. Patients with diagnosis justifying elective delivery are excluded. | | - | lower rates is better performance | NQF | - | Chan [^19^](#_ENREF_19) | Cure | General | - |
| 74 | Epidural steroid injections for back pain | % patients with back pain who received an epidural steroid injection in the absence of radicular pain AND those patients with radicular pain who received an epidural steroid injection without image guidance | | - | lower rates is better performance | NQF | - | Chan [^19^](#_ENREF_19) | Cure | General | - |
| 75 | Surgical timing for back pain | % patients with documentation of red flags who had surgery within the first 6 weeks of back pain onset. | | - | lower rates is better performance | NQF | - | Chan [^19^](#_ENREF_19) | Cure | General | - |
| 76 | Vertebroplasty or kyphoplasty for osteoporotic vertebral fractures | Vertebroplasty/kyphoplasty for vertebral fracture (specified with claim codes) | Patients with osteoporosis | - | - | - | Lit | Schwartz [^3^](#_ENREF_3) | Cure | General | - |
| 77 | Arthroscopic surgery for knee osteoarthritis | Arthroscopic debridement/chondroplasty of the knee (specified with claim codes) | Patients with arthritis | - | - | - | NICE / Lit | Schwartz [^3^](#_ENREF_3) | Cure | General | - |
| 78 | Traction for low back pain | Traction with diagnosis of low back pain | Low back pain diagnosis | - | - | - | IOM | Segal [^18^](#_ENREF_18) | Cure | General | - |
| 79 | Laminectomy or spinal fusion | Laminectomy or spinal fusion | Everyone | those with a clear indication (radicular symptoms* and we will be liberal with this) *symptoms clearly of herniated disc—radicular pain | - | NGC | - | Segal [^18^](#_ENREF_18) | Cure | General | - |
| 80 | Carotid endarterectomy in asymptomatic patients | Carotid endarterectomy for patients without a history of stroke or TIA and without stroke, TIA, or focal neurological symptoms noted in claim (specified with claim codes) | All patients | - | - | - | CW / Lit | Schwartz [^3^](#_ENREF_3) | Cure | General | - |
| 81 | IVC filters to prevent pulmonary embolism | Any IVC filter placement (specified with claim codes) | All patients | - | - | - | Lit | Schwartz [^3^](#_ENREF_3) | Cure | General | - |
| 82 | Percutaneous coronary intervention with balloon angioplasty or stent placement for stable coronary disease | Coronary stent placement or balloon angioplasty for patients with an established diagnosis of ischemic heart disease or angina (≥6 months before the procedure); procedure not associated with an ED visit, which might be indicative of acute coronary syndrome (specified with claim codes) | IHD patients | - | - | - | Lit | Schwartz [^3^](#_ENREF_3) | Cure | General | - |
| 83 | Renal artery angioplasty or stenting | Renal/visceral angioplasty or stent placement (specified with claim codes) | Patients with hypertension | - | - | - | Lit | Schwartz [^3^](#_ENREF_3) | Cure | General | - |
| 84 | Routine monitoring of digoxin in patients with congestive heart failure | Any measure of digoxin with no hospitalizations or ER visits during that year. | All patients* with CHF *will include atrial fibrillation patients as well | - | - | - | NICE | Segal [^18^](#_ENREF_18) | Cure | Specialized | Lab |
| 85 | Homocysteine testing for cardiovascular disease | Homocysteine testing (specified with claim codes) | All patients | No diagnosis for folate or B12 deficiencies in claim and no folate or B12 testing in prior claims | - | - | Lit | Schwartz [^3^](#_ENREF_3) | Cure | General | Lab |
| 86 | Chronic wound care | % patients ≥ 18 with a diagnosis of chronic skin ulcer undergoing debridement with documentation of wound characteristics (including size, nature of wound base tissue, and amount of drainage) prior to debridement. | | - | higher rates is better performance | NGC | - | Chan [^19^](#_ENREF_19) | Cure | General | - |
| 87 | Hysterectomy for benign disease | Any hysterectomy (not specified for malignancy treatment) | All women | those with a malignancy diagnosis | - | NGC | - | Segal [^18^](#_ENREF_18) | Cure | Specialized | - |
| 88 | Don’t recommend percutaneous feeding tubes in patients with advanced dementia | Beneficiaries with two observed dementia diagnoses residing in an institution who received a feeding tube | Institutionalized beneficiaries over age 65 with diagnosed dementia | None | - | - | CW | Colla [^9^](#_ENREF_9) | LTC | Inpatient | - |
| 89 | Inappropriate medications in the elderly | Visits by adults aged ≥ 65 y who received any of 33 potentially inappropriate medications | Visits by adults aged ≥65 y with reported medications | Visits by adults aged ≥65 with diabetes | - | - | Lit | Kale [^25^](#_ENREF_25) | LTC | Outpatient | Pharmaceuticals |
| 90 | Don’t use antipsychotics as first choice to treat behavioral and psychological symptoms of dementia | Beneficiaries who received one or more prescriptions for an antipsychotic following two observed dementia diagnoses | Beneficiaries over age 65 with diagnosed dementia | Severe mental illness during the study period | - | - | CW | Colla [^9^](#_ENREF_9) | LTC | Outpatient | Pharmaceuticals |
| 91 | Cardiac stress imaging (testing in asymptomatic, low-risk patients) | % of all stress SPECT MPI, stress echo, CCTA, and CMR performed in asymptomatic, low CHD risk patients for initial detection and risk assessment | | - | lower rates is better performance | NQF | - | Chan [^19^](#_ENREF_19) | Preventive | - | Imaging |
| 92 | Don’t order cardiac tests on low-risk, asymptomatic patients | Beneficiaries who received a non-indicated cardiac test, echocardiograms, electrocardiograms, advanced cardiac imaging | Low-risk beneficiaries ages 66-80 | Indications of cardiac disease or other conditions that could indicate cardiac testing (e.g. HIV/aids, diabetes, peripheral vascular disease, pulmonary disease, cancer) or use of a prescription drug associated with the above conditions in a calendar year; enrollment in hospice; appropriate clinical indication on testing event claim. | - | - | CW | Colla [^9^](#_ENREF_9) | Preventive | - | Imaging |
| 93 | Endoscopy and polyp surveillance | % patients ≥ 50 receiving a surveillance colonoscopy, with a history of a colonic polyp in previous colonoscopy who had a follow-up interval of > 3 years since their last colonoscopy | | - | higher rates is better performance | NGC / NQF | - | Chan [^19^](#_ENREF_19) | Preventive | - | Imaging |
| 94 | Endoscopy and polyp surveillance | % patients ≥ 50 receiving a screening colonoscopy without biopsy or polypectomy who had a recommend follow-up interval of at least 10 years for repeat colonoscopy. | | - | higher rates is better performance | NGC / NQF | - | Chan [^19^](#_ENREF_19) | Preventive | - | Imaging |
| 95 | Colorectal cancer screening for older patients | Colorectoral cancer screening (colonoscopy, sigmoidoscopy, barium enema, or fecal occult blood testing) for patients aged over 75 y (specified with claim codes) | Patients over 75 | - | - | - | USPTF / Lit | Schwartz [^3^](#_ENREF_3) | Preventive | - | Imaging |
| 96 | Imaging studies in melanoma | % patients with stage 0 or 1A melanoma, without signs or symptoms, for whom no diagnostic imaging studies were ordered | | - | higher rates is better performance | NGC / NQF | - | Chan [^19^](#_ENREF_19) | Preventive | - | Imaging |
| 97 | Bone scan for staging low-risk patients | % patients with a diagnosis of prostate cancer at low risk of recurrence receiving interstitial prostate brachytherapy, OR external beam radiotherapy to the prostate, OR radical prostatectomy, OR cryotherapy who did not have a bone scan performed at any time since the diagnosis of prostate cancer. | | - | higher rates is better performance | NGC / NQF | - | Chan [^19^](#_ENREF_19) | Preventive | - | Imaging |
| 98 | PET, CT, and radionuclide bone scan in individuals with low-risk prostate cancer | PET, CT, or radionuclide bone scan AFTER diagnosis | Men with low risk for prostate CA | - | - | - | CW | Segal [^18^](#_ENREF_18) | Preventive | - | Imaging |
| 99 | Mammography follow-up rates | All patients undergoing diagnostic mammography or breast ultrasound study within 45 days of screening mammography. | All patients receiving a screening mammography study. | No patients. | - | CMS / QualityNet | - | Mathias [^12^](#_ENREF_12) | Preventive | - | Imaging |
| 100 | Mammography follow-up rates | Number of patients who had a diagnostic mammography or an ultrasound of the breast following a screening mammography study within 45 days. | | - | unclear | QualityNet | - | Chan [^19^](#_ENREF_19) | Preventive | - | Imaging |
| 101 | Mammography screening for women aged ≥75 y | Visits by women ages ≥ 75 yr who received a mammogram | Visits by women aged ≥75 y | Visits by women aged ≥75 y with history of breast cancer, breast mass or lump | - | - | UPSTF | Kale [^25^](#_ENREF_25) | Preventive | - | Imaging |
| 102 | Screening for asymptomatic carotid artery stenosis in the general adult population | CPT 93880 or 3100F, ONLY IN outpatient setting (not ER) | All people | ICD-9 codes for: 785.9, 784.2, 362.34, 435.9, 433.10, 342.90, 780.2, 781.3, 437.0 | - | - | UPSTF | Segal [^18^](#_ENREF_18) | Preventive | - | Imaging |
| 103 | Screening for carotid artery disease in asymptomatic adults | Carotid imaging for patients without a history of stroke or TIA and without a diagnosis of stroke, TIA, or focal neurological symptoms in claim (specified with claim codes) | All patients | - | - | - | CW/ USPTF / Lit | Schwartz [^3^](#_ENREF_3) | Preventive | - | Imaging |
| 104 | Screening CME in adults in GME | Visits by adults who present for GME and are ordered a CBC | Visits by adults who present for GME | Visits by adults with cancer, hematologic abnormalities | - | - | UPSTF | Kale [^25^](#_ENREF_25) | Preventive | - | Imaging |
| 105 | Screening ECG in adults in GME | Visits by adults who present for GME and are ordered an ECG | Visits by adults who present for GME | Visits by adults with CAD, arrhytmia, chest pain, HTN, palpitations, dyspnea, or syncope | - | - | UPSTF | Kale [^25^](#_ENREF_25) | Preventive | - | Imaging |
| 106 | Screening UA in adults in GME | Visits by adult men and non-pregnant women who present for GME and are ordered a UA | Visits by adults who present for GME | Visits by adults with urologic disease, pregnancy, or diseases of genital organs | - | - | UPSTF | Kale [^25^](#_ENREF_25) | Preventive | - | Imaging |
| 107 | Screening x-ray in adults in GME | Visits by adults who present for GME and are ordered a chest x-ray | Visits by adults who present for GME | None | - | - | UPSTF | Kale [^25^](#_ENREF_25) | Preventive | - | Imaging |
| 108 | PSA testing for men aged ≥75 y | PSA test for patients aged over 75 y (specified with claim codes) | Men over 75 | - | - | - | USPTF | Schwartz [^3^](#_ENREF_3) | Preventive | - | Lab |
| 109 | Prostate cancer screening in men aged >75 y | Visits by men aged ≥75 yr who are ordered a PSA | Visits by men aged ≥75 y | Visits by adult men aged ≥75 y with prostate cancer | - | - | UPSTF | Kale [^25^](#_ENREF_25) | Preventive | - | Lab |
| 110 | Don’t screen women older than 65 years of age for cervical cancer who have had adequate prior screening and are not otherwise at high risk for cervical cancer | Beneficiaries who received a Pap test | Female beneficiaries at low risk for cervical cancer over age 65 | Gynaecological cancers, HIV/aids, diethylstilbestrol use, or a previous Pap test during the study period | - | NQF | CW | Colla [^9^](#_ENREF_9) | Preventive | - | Lab |
| 111 | Cervical cancer screening in woman aged >65 y | Visits by women ≥ 65 yr who were ordered a Pap test | Visits by women aged ≥65 y | Visits by women aged ≥65 y with cervical cancer, uterine cancer, cervical dysplasia, or vaginal bleeding | - | - | UPSTF | Kale [^25^](#_ENREF_25) | Preventive | - | Lab |
| 112 | Cervical cancer screening for women aged ≥65 y | Screening papanicolaou test for women ages over 65 y (specified with claim codes) | Women over 65 | - | - | - | CW / USPTF | Schwartz [^3^](#_ENREF_3) | Preventive | - | Lab |
| 113 | Follow-up tumor marker studies in asymptomatic women with previously treated breast cancer | 82378 (CEA), 86300 (CA 15-3) (CA 27.29) | Breast cancer is 174.0-174.9 | - | - | - | Lit | Segal [^18^](#_ENREF_18) | Preventive | - | Lab |
| 114 | Don’t perform population based screening for 25-OH-Vitamin D deficiency | Beneficiaries who received a test for vitamin D deficiency | Low-risk beneficiaries over age 65 | Beneficiaries with osteoporosis, fragility fracture, kidney disease, renal dialysis during the same calendar year | - | - | CW | Colla [^9^](#_ENREF_9) | Preventive | - |  |
| 115 | Cancer screening for patients with CKD receiving dialysis | Screening for cancer of the breast, cervix, colon, or prostate for patients with CKD receiving dialysis services. (specified with claim codes) | Patients with CKD | - | - | - | CW / Lit | Schwartz [_ENREF_3^3^](#_ENREF_3) | Preventive | - | Lab/Im |

**Abx: Antibiotics; NGC: National Guideline Clearing House; CMS: Centers for Medicare & Medicaid Services; NQF: National Quality Forum; Lit: Literature; AQC: Alternative Quality Contract (Blue Cross Blue Shield); NICE: National Institute for Clinical Excellence (UK); CADTH: Canadian Agency for Drugs and Technologies in Health; NAEPP: National Asthma Education and Prevention Program; NCQA: National Committee for Quality Assurance; ICSI: Institute for Clinical Systems Improvement; IOM: Institute of Medicine; USPSTF: US Preventive Services Task Force; *if denominator and numerator were not reported separately in the included paper, we reported them acoordingly.**
