# Supplementary material for: Are low-value care measures up to the task? A systematic review of the literature
Source: BMC Health Serv Res. 2016 Aug 18;16:405. doi: 10.1186/s12913-016-1656-3 (PMC4990838; doi:10.1186/s12913-016-1656-3)
Supplement: Additional file 3: — Low-value care recommendations. (DOCX 78 kb) [file 12913_2016_1656_MOESM3_ESM.docx]

Additional file 3. Low-value care recommendations

| **ICHA-HC** |  |  | **First author** | **Low-value care recommendation** |
| --- | --- | --- | --- | --- |
| Alternative | - | - | Elshaug | Acupuncture for Bell’s palsy |
| Alternative | - | - | Elshaug | Acupuncture for depression |
| Alternative | - | - | Elshaug | Acupuncture for induction of labor |
| Alternative | - | - | Elshaug | Acupuncture for irritable bowel syndrome (IBS) |
| Alternative | - | - | Elshaug | Acupuncture for lower urinary tract symptoms (LUTS) in men |
| Alternative | - | - | Elshaug | Acupuncture for peripheral joint osteoarthritis |
| Alternative | - | - | Elshaug | Acupuncture for the management of otitis media with effusion (OME) |
| Alternative | - | - | Elshaug | Acupuncture for uterine fibroids |
| Alternative | - | - | Elshaug | Acupuncture to treat hyperbilirubinemia |
| Alternative | - | - | Elshaug | Acupuncture, acupressure and hypnosis for women in labour |
| Alternative | - | - | Elshaug | Laser acupuncture for carpal tunnel syndrome |
| Cure | Dental | - | Elshaug | Emergency pulpectomy |
| Cure | Dental | - | Elshaug | Porcelain dental crowns |
| Cure | Dental | - | Elshaug | Occlusal adjustment for temporomandibular joint dysfunction |
| Cure | General | - | Chan | More than one ED visit in the last 30 days of life. |
| Cure | General | - | Chan | Routine labor induction |
| Cure | General | - | Chan | Referring OME early in the course of the problem. |
| Cure | General | - | Chan | Potentially preventable ED visits. |
| Cure | General | - | Chan | Routine epidural analgesia. |
| Cure | General | - | Chan | Routine fetal movement counting. |
| Cure | General | - | Chan | Routine vaginal examination to assess gestational age, predict preterm birth, or estimate tight passage during birth. |
| Cure | General | - | Chan | Rupturing membranes (amniotomy) after the start of spontaneous labor whether labor is progressing well or prolonged. |
| Cure | General | - | Elshaug | Caesarean section without medical indication |
| Cure | General | - | Elshaug | Vertebroplasty for painful osteoporotic vertebral factures |
| Cure | General | - | Elshaug | Open surgery for carotid occlusive disease |
| Cure | General | - | Elshaug | Vena Caval Filters for the prevention of pulmonary embolism |
| Cure | General | - | Elshaug | Endovascular repair of infrarenal abdominal aortic aneurysms |
| Cure | General | - | Elshaug | Routine episiotomy associated with spontaneous vaginal birth |
| Cure | General | - | Elshaug | Routine episiotomy associated with vaginal birth following previous third- or fourth degree trauma |
| Cure | General | - | Elshaug | Routine dilation and curettage for missed abortion |
| Cure | General | - | Elshaug | Dilatation and curettage as a diagnostic tool OR therapeutic treatment |
| Cure | General | - | Elshaug | Amnioinfusion for the treatment of women with meconiumstained liquor |
| Cure | General | - | Elshaug | Chest physiotherapy as an adjunctive treatment for adults with pneumonia |
| Cure | General | - | Elshaug | Hospitalization for bed rest in multiple pregnancy |
| Cure | General | - | Elshaug | Neonatal circumcision |
| Cure | General | - | Elshaug | Urinary flow-rate measurement in men with lower urinary tract symptoms (LUTS). |
| Cure | General | - | Hicks | Do not use inferior vena cava filters routinely in patients with acute venous thromboembolism |
| Cure | General | - | Keyhani | Carotid endarterectomy for all indications |
| Cure | General | - | Korenstein | Carotid endarterectomy for carotid stenosis |
| Cure | General | - | Korenstein | IVC filter |
| Cure | General | - | Korenstein | Chiropractic for low back pain |
| Cure | General | - | Korenstein | Nasopharyngeal washings |
| Cure | General | Imaging | Chan | Imaging studies in patients with nonspecific low back pain and no red flags. |
| Cure | General | Imaging | Chan | Performing an imaging stress test as the initial diagnostic test in patients with known or suspected CAD who are able to exercise and have no resting ECG abnormalities that may interfere with interpretation of test results. |
| Cure | General | Imaging | Chan | Coronary angiography in patients with chronic stable angina with well-controlled symptoms on medical therapy or who lack specific high-risk criteria on exercise testing. |
| Cure | General | Imaging | Chan | Obtaining diagnostic images for minor head injuries without loss of consciousness or other risk factors. |
| Cure | General | Imaging | Chan | Imaging for uncomplicated head ache. |
| Cure | General | Imaging | Chan | Imaging studies in patients with recurrent, classic migraine headache and normal findings on neurological examination. |
| Cure | General | Imaging | Chan | Repeat CT scans in patients with functional abdominal pain syndrome if no major changes in clinical findings or symptoms. |
| Cure | General | Imaging | Chan | Sinus imaging for patients with acute rhinosinusitis in absence of predisposing factors for atypical microbial causes. |
| Cure | General | Imaging | Chan | CT or MRI to evaluate simple syncope in patients with normal findings on neurologic examination. |
| Cure | General | Imaging | Chan | Routinely performing ECHO in the evaluation of syncope, unless the history, physical examination, and ECG do not provide a diagnosis or underlying heart disease is suspected. |
| Cure | General | Imaging | Chan | Pre-op chest radiography in the absence of a clinical suspicion for intrathoracic pathology. |
| Cure | General | Imaging | Chan | Performing radionuclide imaging as part of routine follow-up in asymptomatic patients. |
| Cure | General | Imaging | Chan | Routine periodic ECHO in asymptomatic patients with mild aortic stenosis for more frequently than every 3-5 years. |
| Cure | General | Imaging | Chan | Routinely repeat ECHO in asymptomatic patients with mild mitral regurgitation and normal left ventricular size and function. |
| Cure | General | Imaging | Chan | Performing ECHO in asymptomatic patients with innocent-sounding heart murmurs. |
| Cure | General | Imaging | Chan | Electronical fetal monitoring during labor without access to fetal scalp sampling or continuous electronic fetal monitoring. |
| Cure | General | Imaging | Chan | Follow-up imaging for clinically inconsequential adnexal cysts. |
| Cure | General | Imaging | Chan | Home uterine activity monitoring to prevent preterm birth. |
| Cure | General | Imaging | Chan | Routine ultrasound after 24 weeks. |
| Cure | General | Imaging | Chan | Routine ultrasound to estimate fetal size if large baby is suspected. |
| Cure | General | Imaging | Elshaug | Imaging in cases of low back pain* |
| Cure | General | Imaging | Elshaug | Exercise electrocardiogram (ECG) for angina |
| Cure | General | Imaging | Elshaug | Plan x-rays of the skull for diagnosing significant brain injury |
| Cure | General | Imaging | Elshaug | Routine monitoring of bone mineral density after starting bisphosphonate treatment |
| Cure | General | Imaging | Elshaug | Cardiac stress testing on low risk patients before major surgery |
| Cure | General | Imaging | Elshaug | Preoperative chest x-ray |
| Cure | General | Imaging | Elshaug | Cardiotocography for antepartum fetal assessment / antenatal cardiotocography for fetal assessment |
| Cure | General | Imaging | Elshaug | The routine anomaly scan (at 18 weeks 0 days to 20 weeks 6 days) for Down's syndrome screening using soft markers |
| Cure | General | Imaging | Korenstein | Imaging for low back pain |
| Cure | General | Imaging | Korenstein | PT for low back pain |
| Cure | General | Imaging | Korenstein | Preoperative pulmonary function testing |
| Cure | General | Imaging | Onuoha | Preoperative baseline diagnostic cardiac testing (TTE or TEE) or cardiac stress test in asymptomatic stable patients with known cardiac disease (e.g. CAD, vulvular disease) undergoing low-risk or moderate-risk non-cardiac surgery |
| Cure | General | Imaging | Schuur | Do not order magnetic resonance imaging of the lumbar spine for patients with lower back pain without high-risk features. |
| Cure | General | Imaging | Schuur | Do not order CT of the head for patients with mild traumatic head injury who do not meet New Orleans Criteria or Canadian CT Head Rule. |
| Cure | General | Imaging | Schuur | Do not order CT to diagnose pulmonary embolism without first risk stratifying for pulmonary embolism (pretest probability and D-dimer tests if low probability). |
| Cure | General | Imaging | Wood | Patients Who Have No Cardiac History and Good Functional Status Do Not Require Preoperative Stress Testing Before Non-cardiac Thoracic Surgery |
| Cure | General | Imaging | Wood | Before Cardiac Surgery There Is No Need for Pulmonary Function Testing in the Absence of Respiratory Symptoms |
| Cure | General | Lab | Bulger | Do not perform repetitive complete blood count (CBC) and chemistry testing in the face of clinical and lab stability. |
| Cure | General | Lab | Chan | Routine chlamydia screening. |
| Cure | General | Lab | Chan | Routine hepatitis C screening. |
| Cure | General | Lab | Chan | Annual lipid screening for patients without lipid-lowering drug, diet therapy, or reasons for changing lipid profiles. |
| Cure | General | Lab | Chan | Broad spectrum testing rather than focus on likely source. |
| Cure | General | Lab | Chan | Repeat blood tests - creatinine within 10 days. |
| Cure | General | Lab | Chan | Repeat blood tests - ferritin within 6 weeks. |
| Cure | General | Lab | Chan | Repeat blood tests - HDL cholesterol within 6 weeks. |
| Cure | General | Lab | Chan | Repeat blood tests - hemoglobin A1c within 12 weeks |
| Cure | General | Lab | Chan | Repeat blood tests - hemoglobin within 10 days. |
| Cure | General | Lab | Chan | Repeat blood tests - liver function tests (ALT/AST) within 6 weeks. |
| Cure | General | Lab | Chan | Repeat blood tests - sodium within 10 days. |
| Cure | General | Lab | Chan | Repeat blood tests - Thyroid stimulating hormone within 6 weeks. |
| Cure | General | Lab | Chan | Repeat blood tests - total cholesterol within 6 weeks. |
| Cure | General | Lab | Chan | Routine bacterial vaginosis screening. |
| Cure | General | Lab | Chan | Routine preterm labor screening. |
| Cure | General | Lab | Chan | Routine toxoplasmosis screening. |
| Cure | General | Lab | Chan | Serologic testing for Lyme disease in patients with chronic nonspecific symptoms and no clinical evidence of disseminated Lyme disease. |
| Cure | General | Lab | Chan | Serologic testing for suspected early Lyme disease. |
| Cure | General | Lab | Chan | Unnecessary laboratory tests, targeting panels (e.g. thyroid, SMA 20). |
| Cure | General | Lab | Chan | Unnecessary laboratory tests, targeting special testing (e.g. Lyme disease with regional considerations). |
| Cure | General | Lab | Elshaug | Measurement of parathyroid hormone (PTH) levels in people with stage 1, 2, 3A or 3B chronic kidney disease (CKD) |
| Cure | General | Lab | Elshaug | Chlamydia screening in routine antenatal care |
| Cure | General | Lab | Elshaug | Screening for hepatitis C virus in pregnant women |
| Cure | General | Lab | Elshaug | Biochemical tests of placental function for assessment in pregnancy |
| Cure | General | Lab | Elshaug | Blood biochemical testing in children with dehydration |
| Cure | General | Lab | Elshaug | C-reactive protein tests |
| Cure | General | Lab | Elshaug | Factor V Leiden, thrombophilia genetic mutations |
| Cure | General | Lab | Elshaug | Female hormone testing in women with heavy menstrual bleeding (HMB) |
| Cure | General | Lab | Elshaug | Fetal blood sample (FBS) with evidence of acute fetal compromise |
| Cure | General | Lab | Elshaug | Genetic testing of fragile X syndrome - population screen |
| Cure | General | Lab | Elshaug | Human leukocyte antigen (HLA) DQ2/DQ8 testing in the initial diagnosis of coeliac disease |
| Cure | General | Lab | Elshaug | Immunoglobulin G / A (IgG/IgA) anti-gliadin antibody (AGA) test in the diagnosis of coeliac disease. |
| Cure | General | Lab | Elshaug | Liver function tests - Statin therapy |
| Cure | General | Lab | Elshaug | Measurement of calcium levels in people with stage 1, 2, 3A or 3B chronic kidney disease (CKD) |
| Cure | General | Lab | Elshaug | Measurement of phosphate levels in people with stage 1, 2, 3A or 3B chronic kidney disease (CKD) |
| Cure | General | Lab | Elshaug | Microscopy for testing for the presence of hematuria |
| Cure | General | Lab | Elshaug | Nucleic acid amplification tests for diagnosis of Neisseria gonorrhea and Chlamydia trachomatis rectal infections |
| Cure | General | Lab | Elshaug | Preimplantation genetic screening for aneuploidy |
| Cure | General | Lab | Elshaug | Rectal biopsy in suspected Hirschsprungs disease |
| Cure | General | Lab | Elshaug | Routine blood tests in children with fever |
| Cure | General | Lab | Elshaug | Routine screening for preterm labour |
| Cure | General | Lab | Elshaug | Screening for gestational diabetes using fasting plasma glucose, random blood glucose, glucose challenge test and urinalysis for glucose. |
| Cure | General | Lab | Elshaug | Serum cholesterol concentrations in pregnancy |
| Cure | General | Lab | Elshaug | Serum ferritin tests in adults (in patients with chronic fatigue syndrome) |
| Cure | General | Lab | Elshaug | Serum ferritin tests in women with heavy menstrual bleeding |
| Cure | General | Lab | Elshaug | Testing for diarrhea in children |
| Cure | General | Lab | Elshaug | Tests for folate levels in patients with chronic fatigue syndrome |
| Cure | General | Lab | Elshaug | Tests for vitamin B12 deficiency in patients with chronic fatigue syndrome |
| Cure | General | Lab | Elshaug | Troponin levels in acute pulmonary embolism patients |
| Cure | General | Lab | Elshaug | Umbilical cord blood direct antiglobulin test (DAT) (Coombs' test) to predict significant hyperbilirubinemia |
| Cure | General | Lab | Elshaug | Urinary protein measurement in pregnant woman as a predictor of complications of pre-eclampsia |
| Cure | General | Lab | Elshaug | Urine testing in infants and children for urinary tract infection (UTI) |
| Cure | General | Lab | Elshaug | Vertebral biopsy |
| Cure | General | Lab | Rouster-Stevens | Do not test for Lyme disease as a cause of musculoskeletal symptoms without an exposure history and appropriate examination findings. |
| Cure | General | Lab | Schuur | Do not order coagulation studies for patients without hemorrhage or suspected coagulopathy (e.g., with anticoagulation therapy, clinical coagulopathy). |
| Cure | General | Pharmaceuticals | AGS Choosing Wisely Workgroup | Don’t use antimicrobials to treat bacteriuria in older adults unless specific urinary tract symptoms are present. |
| Cure | General | Pharmaceuticals | Bulger | Do not prescribe medications for stress ulcer prophylaxis to medical inpatients unless at high risk for GI complications. |
| Cure | General | Pharmaceuticals | Chan | Routinely prescribe antibiotics for acute mild to moderate sinusitis unless symptoms last for 7 or more days OR symptoms worsen after initial clinical improvement. |
| Cure | General | Pharmaceuticals | Chan | Using non-generic statins when initiating lipid-lowering drug therapy. |
| Cure | General | Pharmaceuticals | Chan | Use of brand over generic Rx for bronchitis, hyperlipidemia, hypo-functioning thyroid gland, ischemic heart disease. |
| Cure | General | Pharmaceuticals | Chan | Diethylstilbestrol to prevent miscarriage. |
| Cure | General | Pharmaceuticals | Chan | Fenfluramine plus phentermine to treat obesity. |
| Cure | General | Pharmaceuticals | Chan | Long-term acid suppression therapy (proton pump inhibitors or histamine-2 receptor antagonists) should be titrated to the lowest effective dose needed to achieve therapeutic goals. |
| Cure | General | Pharmaceuticals | Chan | Routine iron supplementation. |
| Cure | General | Pharmaceuticals | Chan | SSRIs in patients with migraine or tension-type headaches.. |
| Cure | General | Pharmaceuticals | Chan | Thalidomide for sedation in pregnant women. |
| Cure | General | Pharmaceuticals | Chan | Triparanol (MER-29) for cholesterol reduction. |
| Cure | General | Pharmaceuticals | Chan | NSAIDS in individuals with HTN or heart failure or CKD of all causes, including diabetes. |
| Cure | General | Pharmaceuticals | Keyhani | Antibiotics for acute respiratory tract infections |
| Cure | General | Pharmaceuticals | Keyhani | Antibiotics for URI use of diagnostic tests |
| Cure | General | Pharmaceuticals | Korenstein | Antibiotics for URI, acute bronchitis |
| Cure | General | Pharmaceuticals | Korenstein | Acid blockers |
| Cure | General | Pharmaceuticals | Korenstein | Bronchodilators for bronchiolitis obstructive diseases |
| Cure | General | Pharmaceuticals | Quinonez | Do not use bronchodilators in children with bronchiolitis. |
| Cure | General | Pharmaceuticals | Quinonez | Do not use systemic corticosteroids in children under 2 years of age with a lower respiratory tract infection. |
| Cure | General | Pharmaceuticals | Quinonez | Do not treat gastroesophageal reflux in infants routinely with acid suppression therapy. |
| Cure | General | Pharmaceuticals | Wiener | Do not routinely offer pharmacologic treatment with advanced vasoactive agents approved only for the management of pulmonary arterial hypertension to patients with PH resulting from left heart disease or hypoxemic lung diseases (group II or III PH). |
| Cure | General | Pharmaceuticals | Williams | Avoid nonsteroidal anti-inflammatory drugs (NSAIDS) in individuals with hypertension, heart failure, or CKD of all causes, including diabetes. |
| Cure | Specialized | - | Bulger | Avoid transfusions of red blood cells for arbitrary hemoglobin or hematocrit thresholds and in the absence of symptoms or active coronary disease, heart failure, or stroke. |
| Cure | Specialized | - | Bulger | Do not place, or leave in place, urinary catheters for incontinence or convenience or monitoring of output for non–critically ill patients (acceptable indications: critical illness, obstruction, hospice, peri-operatively for <2 days for urologic procedures; use weights instead to monitor diuresis). |
| Cure | Specialized | - | Chan | Chemotherapy in the last 14 days of life. |
| Cure | Specialized | - | Chan | Cancer-directed therapy for solid tumor patients with the following characteristics: low performance status (3 or 4), no benefit from prior evidence-based interventions, not eligible for a clinical trial, and no strong evidence supporting the clinical value of further anticancer treatment. |
| Cure | Specialized | - | Chan | Traction to treat low back pain. |
| Cure | Specialized | - | Chan | Administering ESAs to CKD patients with hemoglobin levels ≥ 10 g/dL without symptoms of anemia. |
| Cure | Specialized | - | Chan | Aggressive interventional procedures. |
| Cure | Specialized | - | Chan | Autologous bone marrow transplant with high-dose chemotherapy for advanced breast cancer. |
| Cure | Specialized | - | Chan | Extracranial-intracranial bypass to reduce the risk of ischemic stroke. |
| Cure | Specialized | - | Chan | Gastric bubble for morbid obesity. |
| Cure | Specialized | - | Chan | Gastric freezing for peptic ulcer disease. |
| Cure | Specialized | - | Chan | Mammary artery ligation for CAD |
| Cure | Specialized | - | Chan | Optic nerve decompression surgery for NAION. |
| Cure | Specialized | - | Chan | Potentially preventable hospital admissions lasting < 24 hours. |
| Cure | Specialized | - | Chan | Radiation therapy for acne |
| Cure | Specialized | - | Chan | Recommending replacement immunoglobin therapy for recurrent infections unless impaired antibody responses to vaccines are demonstrated. |
| Cure | Specialized | - | Chan | Spinal manipulation for treatikng migraine or cluster headaches. |
| Cure | Specialized | - | Chan | Subcutaneous interferon alfa-2a to treat age-related macular degeneration. |
| Cure | Specialized | - | Chan | Supplemental oxygen for healthy premature babies. |
| Cure | Specialized | - | Chan | Unwarranted procedures, targeting knee/hip replacement. |
| Cure | Specialized | - | Chan | White cell stimulating factors for primary prevention of febrile neutropenia for patients < 20% risk for this complication. |
| Cure | Specialized | - | Elshaug | Hysterectomy as a first-line treatment solely for heavy menstrual bleeding |
| Cure | Specialized | - | Elshaug | Surgical approach to hysterectomy for benign gynecological disease, abdominal hysterectomy (AH), vaginal hysterectomy (VH) and laparoscopic hysterectomy (LH) |
| Cure | Specialized | - | Elshaug | Radical prostatectomy |
| Cure | Specialized | - | Elshaug | Radical prostatectomy and external beam radiation therapy |
| Cure | Specialized | - | Elshaug | Upper airway surgery for obstructive sleep apnea syndrome |
| Cure | Specialized | - | Elshaug | Radiotherapy for patients with metastatic spinal cord disease |
| Cure | Specialized | - | Elshaug | Radiotherapy with the intention of preventing metastatic spinal cord compression (MSCC) in patients with asymptomatic spinal metastases. |
| Cure | Specialized | - | Elshaug | Spinal surgery with the intention of preventing metastatic spinal cord compression (MSCC) |
| Cure | Specialized | - | Elshaug | Prostatectomy for early stage prostate cancer |
| Cure | Specialized | - | Elshaug | Active surveillance for men with high-risk localized prostate cancer (active surveillance includes PSA testing and prostate biopsy) |
| Cure | Specialized | - | Elshaug | Surgery for obstructive sleep apnea |
| Cure | Specialized | - | Elshaug | Radiotherapy for patients with metastatic spinal cord compression (MSCC) and planned surgery |
| Cure | Specialized | - | Elshaug | Posterior decompression alone in patients with metastatic spinal cord compression (MSCC). |
| Cure | Specialized | - | Elshaug | Adjuvant radiotherapy with surgery for endometrial cancer |
| Cure | Specialized | - | Elshaug | Anal fistula surgery in patients with inflammatory bowel disease |
| Cure | Specialized | - | Elshaug | Complementary therapies for chronic fatigue syndrome/myalgic encephalomyelitis. |
| Cure | Specialized | - | Elshaug | Conventional photon irradiation in treatment of chordoma |
| Cure | Specialized | - | Elshaug | Coronary stenting (angioplasty) for stable angina and in diabetic patients with multi-vessel disease |
| Cure | Specialized | - | Elshaug | Diagnosis of primary tumor site in metastatic cancer |
| Cure | Specialized | - | Elshaug | Dicectomy |
| Cure | Specialized | - | Elshaug | External fixation versus conservative treatment for distal radial fractures in adults |
| Cure | Specialized | - | Elshaug | Extracorporeal shock wave lithotripsy (ESWL) versus percutaneous nephrolithotomy (PCNL) or retrograde intrarenal surgery (RIRS) for kidney stones |
| Cure | Specialized | - | Elshaug | Extrapleural pneumonectomy for mesothelioma |
| Cure | Specialized | - | Elshaug | Femoral central vein catherization |
| Cure | Specialized | - | Elshaug | Hypothermia for traumatic head injury |
| Cure | Specialized | - | Elshaug | Implantable cardioverter defibrillators |
| Cure | Specialized | - | Elshaug | Interventions for treating acute Achilles tendon ruptures |
| Cure | Specialized | - | Elshaug | Intracavity lavage to reduce the risk of surgical site infection |
| Cure | Specialized | - | Elshaug | IVU for urothelial tumors |
| Cure | Specialized | - | Elshaug | Laparoscopic vs open colposuspension for urinary incontinence in women |
| Cure | Specialized | - | Elshaug | Medial pinning of supracondylar humeral fractures |
| Cure | Specialized | - | Elshaug | Needling for encapsulated trabeculectomy filtering blebs |
| Cure | Specialized | - | Elshaug | Neurosurgical clipping for patients with aneurysmal subarachnoid hemorrhage |
| Cure | Specialized | - | Elshaug | Off-pump heart bypass |
| Cure | Specialized | - | Elshaug | Open total mesorectal excision for rectal cancer |
| Cure | Specialized | - | Elshaug | Pelvic lymphadenectomy for the management of endometrial cancer |
| Cure | Specialized | - | Elshaug | Postoperative radiotherapy for non-small cell lung cancer |
| Cure | Specialized | - | Elshaug | Prophylactic surgical litigation of patent ductus arteriosus for prevention of mortality and morbidity in extremely low birth weight infants |
| Cure | Specialized | - | Elshaug | Radiofrequency facet joint denervation |
| Cure | Specialized | - | Elshaug | Radiotherapy following mastectomy to patients with early invasive breast cancer at low risk of local recurrence |
| Cure | Specialized | - | Elshaug | Radiotherpay for neovascular age-related macular degeneration |
| Cure | Specialized | - | Elshaug | Removal of adenoids |
| Cure | Specialized | - | Elshaug | Rhinomanometry and acoustic rhinometry |
| Cure | Specialized | - | Elshaug | Rubber band ligation versus excisional haemorrhoidectomy for haemorrhoids |
| Cure | Specialized | - | Elshaug | Scalpel versus no-scalpel incision for vasectomy |
| Cure | Specialized | - | Elshaug | Sentinel lymph node biopsy (SLNB) in patients with a preoperative diagnosis of ductal carcinoma in situ (DCIS). |
| Cure | Specialized | - | Elshaug | Standard central venous catheters |
| Cure | Specialized | - | Elshaug | Stem Cell transplantation for AML |
| Cure | Specialized | - | Elshaug | Suprapubic urinary catheter |
| Cure | Specialized | - | Elshaug | Temporary defunctioning stoma in people undergoing anal sphincter repair |
| Cure | Specialized | - | Elshaug | Tension free repair for asymptomatic inguinal hernia |
| Cure | Specialized | - | Elshaug | Total fundoplication for gastroesophageal reflux disease |
| Cure | Specialized | - | Elshaug | Transit studies to diagnose idiopathic constipation |
| Cure | Specialized | - | Elshaug | Transurethral resection of the prostate for symptomatic benign prostatic obstruction |
| Cure | Specialized | - | Elshaug | Tube thoracostomy (TT) in thoracic surgery clinics |
| Cure | Specialized | - | Elshaug | Ultrasound-guided internal jugular (USIJ) versus the subclavian (SC) vein approach for central venous cannulation (CVC) |
| Cure | Specialized | - | Elshaug | UVB therapy for vitiligo |
| Cure | Specialized | - | Elshaug | Whole brain radiotherapy for the treatment of multiple brain metastases |
| Cure | Specialized | - | Halpern | Don't transfuse red blood cells in hemodynamically stable, non-bleeding ICU-patients with a hemoglobin concentration greater than 7 mg/dL. |
| Cure | Specialized | - | Halpern | Don't use parenteral nutrition in adequately nourished critically ill patients within the first seven days of an ICU stay. |
| Cure | Specialized | - | Halpern | Don't deeply sedate mechanically ventilated patients without a specific indication and without daily attempts to lighten sedation. |
| Cure | Specialized | - | Halpern | Don't continue life support for patients at high risk for death or severely impaired functional recovery without offering patients and their families the alternative of care focused entirely on comfort. |
| Cure | Specialized | - | Hicks | In situations where transfusion of RBCs is necessary, transfuse the minimum number of units required to relieve symptoms of anemia or to return the patient to a safe hemoglobin range (7-8 g/dL in stable, non-cardiac in-patients) |
| Cure | Specialized | - | Hicks | Do not administer plasma or prothrombin complex concentrates for non-emergent reversal of vitamin K antagonists (ie, outside of the setting of major bleeding, intracranial hemorrhage, or anticipated emergent surgery) |
| Cure | Specialized | - | Korenstein | Hysterectomy |
| Cure | Specialized | - | Korenstein | Transfusion |
| Cure | Specialized | - | Korenstein | Chemotherapy (colon cancer) |
| Cure | Specialized | - | Korenstein | Coronary artery bypass graft |
| Cure | Specialized | - | Korenstein | Coronary revascularization for coronary artery disease |
| Cure | Specialized | - | Korenstein | Oxygen therapy |
| Cure | Specialized | - | Korenstein | PTCA |
| Cure | Specialized | - | Korenstein | Tympanostomy tubes |
| Cure | Specialized | - | Onuoha | Intraoperative administration of packed red blood cells in a young healthy patient without ongoing blood loss and hemoglobin concentration of >6 g/dL unless symptomatic or hemodynamically unstable |
| Cure | Specialized | - | Onuoha | Intraoperative routine administration of colloid (dextrans, hydroxyethyl starches, albumin) for volume resuscitation without appropriate indications; clinicians should refer to current data for its use in specific populations |
| Cure | Specialized | - | Onuoha | Intraoperative routine use of pulmonary arterial catheter for cardiac surgery in patients with low risk of hemodynamic complications (especially with concomitant use of alternative diagnostic tools, e.g., TTE or TEE) |
| Cure | Specialized | - | Quinonez | Do not use continuous pulse oximetry routinely in children with acute respiratory illness unless they are on supplemental oxygen. |
| Cure | Specialized | - | Wiener | For patients recently discharged on supplemental home oxygen following hospitalization for an acute illness, do not renew the prescription without assessing the patient for ongoing hypoxemia. |
| Cure | Specialized | - | Williams | Don’t administer erythropoiesis-stimulating agents (ESAs) to CKD patients with hemoglobin levels ≥ 10 g/dl without symptoms of anemia. |
| Cure | Specialized | - | Williams | Don’t place peripherally inserted central catheters (PICCs) in stage 3–5 CKD patients without consulting nephrology. |
| Cure | Specialized | Imaging | Bulger | Do not order continuous telemetry monitoring outside of the ICU without using a protocol that governs continuation. |
| Cure | Specialized | Imaging | Chan | Unwarranted diagnostic procedures, targeting uncomplicated chest/thorax CT screening. |
| Cure | Specialized | Imaging | Chan | Performing DEXA screening for osteoporosis in women < 65 years or men <70 years with no risk factors. |
| Cure | Specialized | Imaging | Chan | Performing follow-up imaging studies for incidentally discovered pulmonary nodules ≤ 4 mm in low-risk individuals. |
| Cure | Specialized | Imaging | Chan | Obtaining CT scans in a patient with pneumonia that is confirmed by chest radiography in the absence of complicating clinical or radiographic features. |
| Cure | Specialized | Imaging | Chan | Antinuclear antibody test in patients with nonspecific symptoms or in patients with fibromyalgia. |
| Cure | Specialized | Imaging | Chan | CT for the evaluation of suspected appendicitis in children until after ultrasound has been considered as an option. |
| Cure | Specialized | Imaging | Chan | Performing follow-up surveillance examination in < 3 years if patient had a second endoscopy that confirms the absence of dysplasia on biopsy. |
| Cure | Specialized | Imaging | Chan | Performing imaging studies as the initial diagnostic test in patients with low pretest of VTE. |
| Cure | Specialized | Imaging | Chan | Performing pre-discharge chest radiography for hospitalized patients with community-acquired pneumonia who are making satisfactory clinical recovery. |
| Cure | Specialized | Imaging | Chan | Repeat imaging studies (< 60 days since prior test). |
| Cure | Specialized | Imaging | Chan | Repeating screening ultrasonography for following a negative study. |
| Cure | Specialized | Imaging | Chan | Unwarranted diagnostic procedures, targeting bone or joint x-ray prior to conservative therapy, without red flags. |
| Cure | Specialized | Imaging | Chan | Unwarranted diagnostic procedures, targeting endoscopy. |
| Cure | Specialized | Imaging | Chan | Unwarranted diagnostic procedures, targeting chest x-ray, preoperative, on admission or routine monitoring. |
| Cure | Specialized | Imaging | Elshaug | Chest radiograph in acute respiratory infections |
| Cure | Specialized | Imaging | Elshaug | Routine chest x-rays on children with fever (without features of serious illness) |
| Cure | Specialized | Imaging | Elshaug | Halter monitoring (24 hour ECG) in young patients with palpitations and history indicating ectopic beats |
| Cure | Specialized | Imaging | Elshaug | Computerized tomography (CT) of the pelvis in men with low- or intermediate-risk localized prostate cancer |
| Cure | Specialized | Imaging | Elshaug | Chest x-ray in children with symptoms and signs suggesting pneumonia |
| Cure | Specialized | Imaging | Elshaug | Abdominal ultrasound to diagnose idiopathic constipation |
| Cure | Specialized | Imaging | Elshaug | Angiography in lower limb vascular trauma patients |
| Cure | Specialized | Imaging | Elshaug | Auditory brainstem responses for diagnosing CFS |
| Cure | Specialized | Imaging | Elshaug | Chest x-ray for diagnosis of acute coronary syndrome |
| Cure | Specialized | Imaging | Elshaug | CT or MRI in primary aldosteronism |
| Cure | Specialized | Imaging | Elshaug | CT or ultrasound to diagnose appendicitis |
| Cure | Specialized | Imaging | Elshaug | CT scans (head) in children with low risk of clinically important brain injuries after trauma |
| Cure | Specialized | Imaging | Elshaug | Cystoscopy for men with uncomplicated lower urinary tract symptoms (LUTS). |
| Cure | Specialized | Imaging | Elshaug | Endoscopic retrograde cholangiopancreatiography in acute gallstone pancreatitis without cholangitis |
| Cure | Specialized | Imaging | Elshaug | Fluorimetry or endoscopy to assess dysphasia |
| Cure | Specialized | Imaging | Elshaug | Imaging of the upper urinary tract in men with uncomplicated lower urinary tract symptoms (LUTS)> |
| Cure | Specialized | Imaging | Elshaug | Inappropriate indication for upper endoscopy |
| Cure | Specialized | Imaging | Elshaug | Mammography of the ipsilateral soft tissues after mastectomy |
| Cure | Specialized | Imaging | Elshaug | Plain abdominal radiograph to diagnose idiopathic constipation in children and young people |
| Cure | Specialized | Imaging | Elshaug | Plain radiographs of the spine to make or to exclude the diagnosis of spinal metastases or metastatic spinal cord compression (MSCC). |
| Cure | Specialized | Imaging | Elshaug | Routine daily chest radiographs in intensive care |
| Cure | Specialized | Imaging | Elshaug | Routine imaging of the spine in patients with a previous diagnosis of malignancy |
| Cure | Specialized | Imaging | Elshaug | Routine screening for cardiac anomalies using nuchal translucency |
| Cure | Specialized | Imaging | Elshaug | Routine spinal magnetic resonance imaging (MRI) for all men with hormone-refractory prostate cancer and known bone metastases |
| Cure | Specialized | Imaging | Elshaug | Routine ultrasound in infants or children for (UTI) |
| Cure | Specialized | Imaging | Elshaug | Saline infusion sonography as a first-line diagnostic tool |
| Cure | Specialized | Imaging | Hicks | Limit surveillance CT scans in asymptomatic patients after curative-intent treatment for aggressive lymphoma |
| Cure | Specialized | Imaging | Keyhani | Coronary angiography for acute myocardial infarction |
| Cure | Specialized | Imaging | Keyhani | Coronary angiography - for all indications |
| Cure | Specialized | Imaging | Keyhani | Radionuclide myocardial perfusion imaging |
| Cure | Specialized | Imaging | Korenstein | Coronary angiography for myocardial infarction, coronary artery disease |
| Cure | Specialized | Imaging | Korenstein | Radiographs in acute respiratory illnesses for bronchiolitis or croup, asthma |
| Cure | Specialized | Imaging | Korenstein | CT scan for epilepsy |
| Cure | Specialized | Imaging | Korenstein | Endoscopy (upper) for bleeding (upper), peptic ulcer disease |
| Cure | Specialized | Imaging | Quinonez | Do not order chest radiographs in children with asthma or bronchiolitis. |
| Cure | Specialized | Imaging | Rouster-Stevens | Do not routinely perform surveillance joint radiographs to monitor JIA disease activity. |
| Cure | Specialized | Imaging | Schuur | Do not order computed tomography (CT) of the cervical spine for patients after trauma who do not meet the National Emergency X-ray Utilization Study (NEXUS) low-risk criteria9 or the Canadian C-Spine Rule. |
| Cure | Specialized | Imaging | Wiener | Do not perform CT scan surveillance for evaluation of indeterminate pulmonary nodules at more frequent intervals or for a longer period of time than recommended by established guidelines. |
| Cure | Specialized | Imaging | Wiener | Do not perform chest CT angiography to evaluate for possible PE in patients with a low clinical probability and negative results of a highly sensitive D -dimer assay. |
| Cure | Specialized | Imaging | Wood | Do Not Initiate Routine Evaluation of Carotid Artery Disease Before Cardiac Surgery in the Absence of Symptoms or Other High-Risk Criteria |
| Cure | Specialized | Imaging | Wood | Do Not Perform a Routine Pre-discharge Echocardiogram After Cardiac Valve Replacement Surgery |
| Cure | Specialized | Imaging | Wood | Patients With Suspected or Biopsy Proven Stage I NSCLC Do Not Require Brain Imaging Before Definitive Care in the Absence of Neurologic Symptoms |
| Cure | Specialized | Lab | Chan | Performing unproven diagnostic tests (e.g. IgG or IGE) in the evaluation of allergy |
| Cure | Specialized | Lab | Chan | Performing follow-up complete blood counts, blood chemistry studies, tumor marker studies, chest radiography, or imaging studies other than appropriate breast imaging in asymptomatic women with previously treated breast cancer |
| Cure | Specialized | Lab | Chan | Measuring brain natriuretic peptide in the initial evaluation of patients with typical findings of heart failure. |
| Cure | Specialized | Lab | Chan | Pre-op coagulation tests when risk or predisposing factors for bleeding and history of abnormal bleeding are absent. |
| Cure | Specialized | Lab | Chan | Routine pre-op laboratory tests in otherwise healthy patients undergoing elective surgery. |
| Cure | Specialized | Lab | Chan | Routinely do diagnostic testing in patients with chronic urticaria |
| Cure | Specialized | Lab | Elshaug | Tissue biopsy to reassess HER2 status |
| Cure | Specialized | Lab | Elshaug | Tissue biopsy to reassess ER status |
| Cure | Specialized | Lab | Elshaug | Assessing progesterone receptor status of tumors in patients with invasive breast cancer |
| Cure | Specialized | Lab | Elshaug | Inflammatory markers for prediction of recurrent stroke |
| Cure | Specialized | Lab | Elshaug | Measurement of alfa-fetoprotein in alpha-fetoprotein producing gastric cancers |
| Cure | Specialized | Lab | Elshaug | Measurement of bilirubin levels in babies who are not visibly jaundiced |
| Cure | Specialized | Lab | Elshaug | Mortality markers in end stage renal disease |
| Cure | Specialized | Lab | Elshaug | Troponin Tests for evaluation of heart attack/heart injury |
| Cure | Specialized | Lab | Elshaug | Uro4 HB&L system for the rapid diagnosis of lower respiratory tract infections in intensive care units |
| Cure | Specialized | Lab | Halpern | Don't order diagnostic tests at regular intervals (such as every day), but rather in response to specific clinical questions. |
| Cure | Specialized | Lab | Hicks | Do not test for thrombophilia in adult patients with venous thromboembolism occurring in the setting of major transient risk factors (surgery, trauma, or prolonged immobility) |
| Cure | Specialized | Lab | Korenstein | Tumor markers |
| Cure | Specialized | Lab | Onuoha | Preoperative baseline laboratory studies (CBC, BMP or CMP, coagulation studies) in healthy patients without significant systemic disease (ASA I or II), when blood loss (or fluid shifts) is expected to be minimal |
| Cure | Specialized | Lab | Rouster-Stevens | Do not order autoantibody panels unless positive ANAs and evidence of rheumatic disease |
| Cure | Specialized | Lab | Rouster-Stevens | Do not repeat a confirmed positive ANA in patients with established JIA or SLE. |
| Cure | Specialized | Lab | Rouster-Stevens | Do not perform methotrexate toxicity labs more often than every 12 weeks when patients are on stable doses. |
| Cure | Specialized | Lab/Im | Korenstein | Post-cancer surveillance |
| Cure | Specialized | Pharmaceuticals | Chan | Chelation therapy to prevent or reverse atherosclerosis. |
| Cure | Specialized | Pharmaceuticals | Chan | Hydralazine for CHF. |
| Cure | Specialized | Pharmaceuticals | Chan | Lidocaine to prevent arrhythmia and sudden death in AMI. |
| Cure | Specialized | Pharmaceuticals | Chan | Quinidine for suppressing recurrences of atrial fibrillation. |
| LTC | Day | - | Elshaug | Lower-extremity arteriovenous access for hemodialysis |
| LTC | Day | - | Williams | Don’t initiate chronic dialysis without ensuring a shared decision-making process between patients, their families, and their physicians. |
| LTC | Inpatient | - | AGS Choosing Wisely Workgroup | Don’t recommend percutaneous feeding tubes in patients with advanced dementia; instead, offer oral assisted feeding. |
| LTC | Inpatient | - | AGS Choosing Wisely Workgroup | Avoid using physical restraints to manage behavioral symptoms of hospitalized older adults with delirium |
| LTC | Inpatient | Pharmaceuticals | AGS Choosing Wisely Workgroup | Don’t use benzodiazepines or other sedative–hypnotics in older adults as first choice for insomnia, agitation, or delirium. |
| LTC | Inpatient | Pharmaceuticals | AGS Choosing Wisely Workgroup | Avoid using prescription appetite stimulates or high-calorie supplements for treatment of anorexia or cachexia in older adults; instead optimize social supports, providing feeding assistance, and clarify patient goals and expectations |
| LTC | Inpatient | Pharmaceuticals | AGS Choosing Wisely Workgroup | Don't prescribe a medication without conducting a drug regimen review |
| LTC | Inpatient | Pharmaceuticals | AGS Choosing Wisely Workgroup | Don't prescribe cholinesterase inhibitors for dementia without periodic assessment for perceived cognitive benefits and adverse gastrointestinal effects. |
| LTC | Outpatient | - | AGS Choosing Wisely Workgroup | Don’t use antipsychotics as first choice to treat behavioral and psychological symptoms of dementia. |
| LTC | Outpatient | - | Chan | Monitoring patients with asthma or COPD by using full pulmonary function testing that includes lung volumes and diffusing capacity, rather than spirometry alone (or peak expiratory flow rate monitoring in asthma). |
| LTC | Outpatient | - | Elshaug | Spirometry during COPD exacerbation and treatment monitoring |
| LTC | Outpatient | Lab | Elshaug | Frequent monitoring HbA1C levels in adults with diabetes |
| LTC | Outpatient | Pharmaceuticals | AGS Choosing Wisely Workgroup | Avoid using medications to achieve hemoglobin A1c <7.5% in most adults age 65 and older; moderate control is generally better. |
| LTC | Outpatient | Pharmaceuticals | Amos | Amitryptiline in ≥65 year olds |
| LTC | Outpatient | Pharmaceuticals | Amos | Chlorpropamide in ≥65 year olds |
| LTC | Outpatient | Pharmaceuticals | Amos | Citalopram > 20 mg / day in ≥65 year olds |
| LTC | Outpatient | Pharmaceuticals | Amos | Clonidine (oral) in ≥65 year olds |
| LTC | Outpatient | Pharmaceuticals | Amos | Disopyramide in ≥65 year olds |
| LTC | Outpatient | Pharmaceuticals | Amos | Escitalopram >10 mg/day in ≥65 year olds |
| LTC | Outpatient | Pharmaceuticals | Amos | Indomethacin in ≥65 year olds |
| LTC | Outpatient | Pharmaceuticals | Amos | Ketorolac max 2 days in ≥65 year olds |
| LTC | Outpatient | Pharmaceuticals | Amos | Methyldopa in ≥65 year olds |
| LTC | Outpatient | Pharmaceuticals | Amos | Nifedipine (short acting) in ≥65 year olds |
| LTC | Outpatient | Pharmaceuticals | Amos | NSAID's > 15 days in ≥65 year olds |
| LTC | Outpatient | Pharmaceuticals | Amos | Oestrogen in ≥65 year olds |
| LTC | Outpatient | Pharmaceuticals | Amos | Orphenadrine in ≥65 year olds |
| LTC | Outpatient | Pharmaceuticals | Amos | Pentazocine in ≥65 year olds |
| LTC | Outpatient | Pharmaceuticals | Amos | Spironolactone >25 mg/day in ≥65 year olds |
| LTC | Outpatient | Pharmaceuticals | Amos | Testosterone in ≥65 year olds |
| LTC | Outpatient | Pharmaceuticals | Chan | Inappropriate medication use, targeting polypharmacy (for multiple chronic conditions; of antipsychotics). |
| LTC | Outpatient | Pharmaceuticals | Chan | Overuse or early use of third-line treatment, for example Avandia for diabetes. |
| Preventive | - | - | Chan | Screening for COPD with spirometry in individuals without respiratory symptoms |
| Preventive | - | - | Elshaug | Spirometry for COPD screening |
| Preventive | - | Imaging | AGS Choosing Wisely Workgroup | Don't recommend screening for breast or colectoral cancer, nor prostate cancer (with the prostate-specific antigen test) without considering life expectancy and the risks of testing, over-diagnosis and overtreatment |
| Preventive | - | Imaging | Chan | Coronary heart disease screening using ECG, exercise treadmill test (ETT), electron beam CT in low-risk adults |
| Preventive | - | Imaging | Chan | Obtaining ECGs to screen for cardiac disease in patients at low to average risk for CAD. |
| Preventive | - | Imaging | Chan | Obtaining exercise ECG for screening in low-risk asymptomatic adults. |
| Preventive | - | Imaging | Chan | Ordering annual ECG or any other cardiac screening for asymptomatic, low-risk patients. |
| Preventive | - | Imaging | Chan | Screening for colectoral cancer in adults older than 75 years or in adults with a life expectancy of less than 10 years. |
| Preventive | - | Imaging | Chan | Repeating colonoscopy within 5 years in: (a) asymptomatic patients with low-risk adenomas, (b) patients with one or two small (< 1 cm) adenomatous polyps, without high-grade dysplasia, completely removed via high-quality colonoscopy. |
| Preventive | - | Imaging | Chan | Carotid artery stenosis screening in general adult population |
| Preventive | - | Imaging | Chan | Repeat cardiac studies within a 3-month period. |
| Preventive | - | Imaging | Chan | Using MRI rather than mammography as the breast cancer screening test of choice for average-risk women. |
| Preventive | - | Imaging | Elshaug | Fecal occult blood screening for colorectal cancer |
| Preventive | - | Imaging | Keyhani | Surveillance endoscopy |
| Preventive | - | Imaging | Korenstein | Cardiac stress test |
| Preventive | - | Imaging | Korenstein | Fecal occult blood screening for colorectal cancer |
| Preventive | - | Imaging | Korenstein | Echocardiogram |
| Preventive | - | Imaging | Korenstein | Periodic health examination: electrocardiogram |
| Preventive | - | Imaging | Korenstein | Colonoscopy for colon cancer screening and follow-up |
| Preventive | - | Imaging | Korenstein | Periodic health examination: chest radiography |
| Preventive | - | Imaging | Wiener | Do not perform CT scan screening for lung cancer among patients at low risk for lung cancer. |
| Preventive | - | Lab | Chan | Prostate cancer screening in males ≥ 75 years or with a life expectancy of < 10 years. |
| Preventive | - | Lab | Chan | Pap tests in females <21 years or post-hysterectomy for benign disease; cervical cancer screening in average in average to low-risk females ≥ 65 years or post-hysterectomy for benign disease. |
| Preventive | - | Lab | Chan | Using CA-125 antigen levels to screen woman for ovarian cancer in the absence of increased risk. |
| Preventive | - | Lab | Chan | Blood chemistry panels or urinalyses for screening in asymptomatic, healthy adults. |
| Preventive | - | Lab | Chan | BRCA mutation testing for breast and ovarian cancer in low-risk females. |
| Preventive | - | Lab | Chan | Screening low-risk individuals for hepatitis B virus infection. |
| Preventive | - | Lab | Elshaug | Prostate specific antigen (PSA) testing |
| Preventive | - | Lab | Elshaug | The routine measurement of vitamin D levels in stage 1, 2, 3A or 3B chronic kidney disease (CKD) is not recommended |
| Preventive | - | Lab | Elshaug | Chlamydia screening in under 25 year olds |
| Preventive | - | Lab | Korenstein | Prostate-specific antigen :prostate cancer screening |
| Preventive | - | Lab | Korenstein | Papanicolaou test: cervical cancer screening |
| Preventive | - | Lab | Korenstein | Periodic health examination: urinalysis |
| Preventive | - | Lab/Im | Chan | Routine cancer screening for dialysis patients with limited life expectancies without signs or symptoms. |
| Preventive | - | Lab/Im | Williams | Don’t perform routine cancer screening for dialysis patients with limited life expectancies without signs or symptoms. |
| Rehabilitative | - | - | Elshaug | CBT for schizophrenia, bipolar disorder and major depression |
| Rehabilitative | - | - | Elshaug | Electroconvulsive therapy (ECT) for people with moderate depression |
| Rehabilitative | - | - | Elshaug | Social skills training (as a specific intervention) to people with schizophrenia |
| Rehabilitative | - | - | Elshaug | Structural neuroimaging techniques (either magnetic resonance imaging (MRI) or computed axial tomography (CT) scanning) for the management of first-episode psychosis |

**Lab.: Laboratory; Im.: Imaging**
